# Impact of red blood cell transfusion on clinical outcomes after cardiovascular surgery: a reconstructed time-to-event meta-analysis

**DOI:** 10.1186/s13019-026-04549-0

**Published:** 2026-07-29

**Authors:** Ogechukwu Samuel Obi, Vanisha Kumar, Gokul Rajith, Matheus S. Avila, Omer Farooq Mohammed, Asad Iqbal, Muhammad Khan, Uchenna Diane Nweze, Kamil Ahmad Kamil, Alida Podrumar, Michel Pompeu Sá

**Affiliations:** 1 Lexington Medical Center, West Columbia, SC USA; 2 All India Institute of Medical Sciences, Guwahati, Assam India; 3 Lusíada University Center, Santos, São Paulo, Brazil; 4 Osmania Medical College, Hyderabad, Telangana India; 5 Bacha Khan Medical College, Mardan, Pakistan; 6 Kaiser Permanente Fontana Medical Center, Fontana, CA USA; 7 Chief, Department of Internal Medicine, Nasrat Bashir Curative Hospital, Kandahar, Afghanistan; 8 Division of Hematology and Oncology, Nassau University Medical Center, East Meadow, NY USA; 9 Department of Cardiothoracic Surgery, Cleveland Clinic Florida, Weston, FL USA

**Keywords:** Blood transfusion, CABG, Cardiovascular surgery, Mortality

## Abstract

**Background:**

The impact of perioperative red blood cell transfusion on long-term outcomes after cardiovascular surgery remains controversial. This meta-analysis of reconstructed time-to-event data aimed to evaluate the association between red blood cell transfusion and clinical outcomes in adult patients undergoing cardiovascular surgery.

**Methods:**

PubMed, Scopus, Embase, and the Cochrane Library were systematically searched for studies comparing perioperative red blood cell transfusion versus no transfusion in adults undergoing cardiovascular surgery. The primary endpoint was all-cause mortality at short- and long-term follow-up. A pairwise random-effects meta-analysis was done for 30-day mortality and other secondary outcomes, pooling risk ratios (RR) with 95% confidence intervals (CIs). Time-to-event data were reconstructed from published Kaplan–Meier curves to perform landmark analysis and estimate restricted mean survival time (RMST) differences between red blood cell transfused and non-transfused groups. Cox frailty regression analysis was used to obtain hazard ratios (HR). Meta-regression analyses were performed to explore sources of heterogeneity, and the certainty of evidence was assessed using the GRADE framework.

**Results:**

This systematic review and meta-analysis included 15 observational studies (13 retrospective and 2 prospective) comparing the impact of red blood transfusion on cardiovascular outcomes in adult patients after cardiovascular surgery. Red blood cell transfusion was associated with a higher incidence of 30-day mortality (RR = 2.57, 95% CI [1.96, 3.38], I² = 0.0%, *p* < 0.001), long-term all-cause mortality (HR 1.79 (95% CI [1.75, 1.83]; *p* < 0.001)*, atrial fibrillation (RR 1.57 95% CI [1.21, 2.04]; *p* < 0.001), acute kidney injury (RR 4.52 95% CI [2.32, 8.80]; *p* < 0.001), myocardial infarction (RR 2.32 95% CI [1.60, 3.37]; *p* < 0.001), pneumonia (RR 5.10 95% CI [3.04, 8.57]; *p* < 0.001), reoperation for bleeding (RR 12.61 95% CI [7.99, 19.90]; *p* < 0.001), sepsis (RR 7.49 95% CI [2.64, 21.26]; *p* < 0.001), and stroke (RR 2.54 95% CI [2.04, 3.16]; *p* < 0.001).

**Conclusions:**

These findings suggest that perioperative red blood cell transfusion was associated with higher rates of mortality and adverse outcomes; however, these findings should be interpreted cautiously because of residual confounding, baseline differences between groups, and the observational nature of the included studies.

**Supplementary Information:**

The online version contains supplementary material available at https://doi.org/10.1186/s13019-026-04549-0.

## Introduction

Blood transfusion has been a cornerstone of modern medical practice for over a century, commonly used to treat anemia, manage hemorrhagic shock, and optimize oxygen delivery in critically ill patients. Despite these potential benefits, transfusion is associated with several risks, including transfusion-related acute lung injury, immunomodulatory effects, circulatory overload, and impaired microcirculatory oxygen delivery [1–4]. In cardiovascular surgery, anemia and low hemoglobin levels have been linked to impaired oxygen transport and increased mortality risk [5].

Previous studies have demonstrated that both perioperative anemia and transfusion of blood products are independently associated with increased morbidity and mortality following cardiac surgery [6–8]. Furthermore, transfusion has been correlated with higher rates of postoperative complications, including renal dysfunction, pneumonia, wound infection, sepsis, and in-hospital mortality [9–13]. Although several randomized controlled trials have evaluated restrictive versus liberal transfusion strategies in critically ill and cardiac surgical populations, most have primarily focused on short-term outcomes, leaving the long-term cardiovascular and survival impact of transfusion incompletely characterized [14, 15].

Prior meta-analyses have reported a significant association between perioperative transfusion and long-term mortality after cardiac surgery [16, 17]. However, these findings may be influenced by residual confounding factors such as surgical complexity, transfusion timing, and transfusion dose. Additionally, some earlier meta-analyses included studies with overlapping populations or lacked direct comparisons between transfused and non-transfused patients, potentially limiting the robustness of the conclusions.

The long-term impact of perioperative RBC transfusion after cardiovascular surgery remains uncertain, particularly because previous studies have been limited by heterogeneous populations, inconsistent adjustment strategies, and a focus on short-term outcomes. Therefore, we conducted a meta-analysis to evaluate the association between RBC transfusion and short- and long-term clinical outcomes in adults undergoing cardiovascular surgery. To better characterize long-term survival, we performed a reconstructed time-to-event analysis using published Kaplan–Meier data. This approach was necessary to provide a more comprehensive assessment of survival outcomes and to address important gaps in the current evidence base.

## Methods

Methodology and reporting followed the Cochrane Collaboration Handbook for Systematic Reviews of Interventions and the Preferred Reporting Items for Systematic Reviews and Meta-Analysis (PRISMA) Statement Guidelines [18, 19]. This review protocol was prospectively registered in the International Prospective Register of Systematic Reviews (PROSPERO) under registration number [CRD420261308403].

### Eligibility criteria

This meta-analysis strictly comprised studies that fulfilled the following eligibility criteria: (i) observational studies, and (ii) those comparing the effects of red blood cell transfusion versus no transfusion on cardiovascular outcomes in adult patients following cardiovascular surgery. Excluded studies included in vitro analyses, case reports, animal studies, conference abstracts lacking complete data, review articles, studies with incomplete outcome reporting, studies with overlapping populations, studies not directly comparing transfusion versus no transfusion arms. Non-English studies were also excluded, as a preliminary search indicated that fewer than 5% of potentially eligible studies were published in non-English languages, and none provided extractable outcome data. Including these non-English studies would not have significantly altered the pooled estimates but could have introduced translation bias and inconsistencies in the data. Only studies published in English were included. This restriction was applied a priori for feasibility and consistency of data extraction and interpretation. Nevertheless, the exclusion of non-English publications may have introduced language bias and should be considered when interpreting the findings.

### Search strategy and data collection

An extensive literature search was conducted using PubMed, Embase, Scopus, and the Cochrane Library, utilizing the following search terms: red blood cell transfusion, transfusion, red blood cells, cardiovascular surgery, coronary artery bypass graft (CABG), cardiac surgery, vascular surgery, valvular surgery, cardiac operations, and cardiac valve operations, among others. For detailed information, please refer to the Supplementary Appendix. The search covered studies published up until December 2025, beginning from the inception date of each database. A re-evaluation of the search was performed prior to the final analysis.

Articles retrieved from each database were exported to Rayyan. To prevent double-counting, studies were screened for overlapping or duplicate cohorts, particularly those derived from large national registries and multicenter databases. Potential cohort overlap was assessed by comparing data sources, enrollment periods, participating institutions, geographic regions, and inclusion criteria. Attention was paid to studies derived from the Society of Thoracic Surgeons (STS) database and other large registries. When overlap was suspected, the study with the most comprehensive dataset, longest follow-up, or most relevant outcome reporting was retained, while potentially overlapping reports were excluded to ensure that each patient population was included only once.

Duplicate records were eliminated, and two authors (O.S.O and U.D.N) independently reviewed all titles and abstracts. Any discrepancies among the reviewers were resolved by an independent fourth author (MP. Sá).

Articles meeting inclusion criteria underwent full-text review, with any disputes addressed by the independent third author. Data were extracted by two independent reviewers from the titles, abstracts, and full-text articles and compiled into an electronic spreadsheet. In cases of conflict, the final decision rested with a third author (MP. Sá).

### Outcome measures

Clinical outcomes were extracted from each study included in the analysis. The primary outcomes focused on 30-day mortality and long-term mortality rates. Short-term mortality was defined as all-cause death occurring within 30 days of the index procedure or hospitalization. Long-term mortality was defined as all-cause mortality beyond 30 days, quantified using the adjusted hazard ratio corresponding to the longest available follow-up within each study. Secondary outcomes encompassed atrial fibrillation (Afib), acute kidney injury (AKI), myocardial infarction (MI), pneumonia, reoperation due to bleeding, sepsis, sternal infection, and stroke. Subgroup analyses were performed based on study design, type of cardiac surgery, and blood transfusion dose.

### Risk of bias assessment

The evaluation of bias in the studies was conducted using The Risk Of Bias In Non-randomized Studies of Interventions (ROBINS-I) tool [20]. We reviewed seven domains: confounding, selection bias, bias in the measurement and classification of interventions, bias due to deviations from intended interventions, bias stemming from missing data, bias in the measurement of outcomes, and bias in the selection of reported results. Each domain was assessed and categorized with a risk level of low, moderate, severe, or critical. This process was carried out by two independent authors (O.F.M and O.S.O), with a senior author (MP. Sá) mediating and resolving any conflicts that arose.

The quality assessment results were presented as a plot utilizing the Robin tool [21]. Additionally, the GRADE approach was employed to evaluate the certainty of the evidence [22].

### Statistical analysis

We evaluated treatment effects on dichotomous outcomes by calculating effect-size risk ratios (RRs) or hazard ratios (HRs), as reported in the studies, with their 95% confidence intervals (CIs), and considered p-values < 0.05 indicative of statistical significance. Prediction intervals (PIs) were utilized to estimate the range of values anticipated in future trials. The Mantel-Haenszel (MH) test, combined with a random-effects model, was employed to assess heterogeneity for each outcome. For quantitative outcomes, we compared using the mean difference (MD) with a random-effects inverse-variance model, and results were presented with forest plots.

Heterogeneity among studies was assessed using Cochran’s Q test and I² statistics, adhering to Cochran’s predefined thresholds: 0–40% indicating low heterogeneity, 30–60% moderate heterogeneity, 50–90% substantial heterogeneity, and 75–100% considerable heterogeneity. Sensitivity analyses, encompassing subgroup analyses and leave-one-out sensitivity analysis, were conducted as part of this meta-analysis.

Statistical analysis of the outcomes was performed using R software version 4.3.2 (The R Project for Statistical Computing, Vienna, Austria). The analysis relied on three statistical packages: meta, metafor, ggplot2, and dmetar [23–26]. The methods of Luo and Wan were applied for converting the median to the mean [27, 28].

### Time to event analysis

A meta-analysis of Kaplan–Meier–derived individual patient data was performed for long-term outcomes using the IPD from KM reconstruction method [29]. Proportional hazards assumptions were evaluated visually using Schoenfeld residual plots and formally tested with the Grambsch–Therneau test. Hazard ratios (HRs) with 95% confidence intervals were estimated using Cox frailty regression models, with treatment group (blood transfusion versus no transfusion) included as a fixed effect and individual studies modeled as random effects through a frailty term to account for between-study heterogeneity. When proportional hazards assumptions were violated, restricted mean survival time differences (∆RMST) were calculated from reconstructed datasets and pooled using a random-effects meta-analysis, as recommended for time-to-event analyses when hazards are non-proportional [30]. Landmark analysis was undertaken to distinguish early and late treatment effects when visual inspection of KM curves suggested potential temporal differences in hazard and the proportional hazard assumption is violated. Stratified log-rank tests were used to compare survival distributions while accounting for study-level stratification.

## Results

### Study selection

**Fig. 1:**
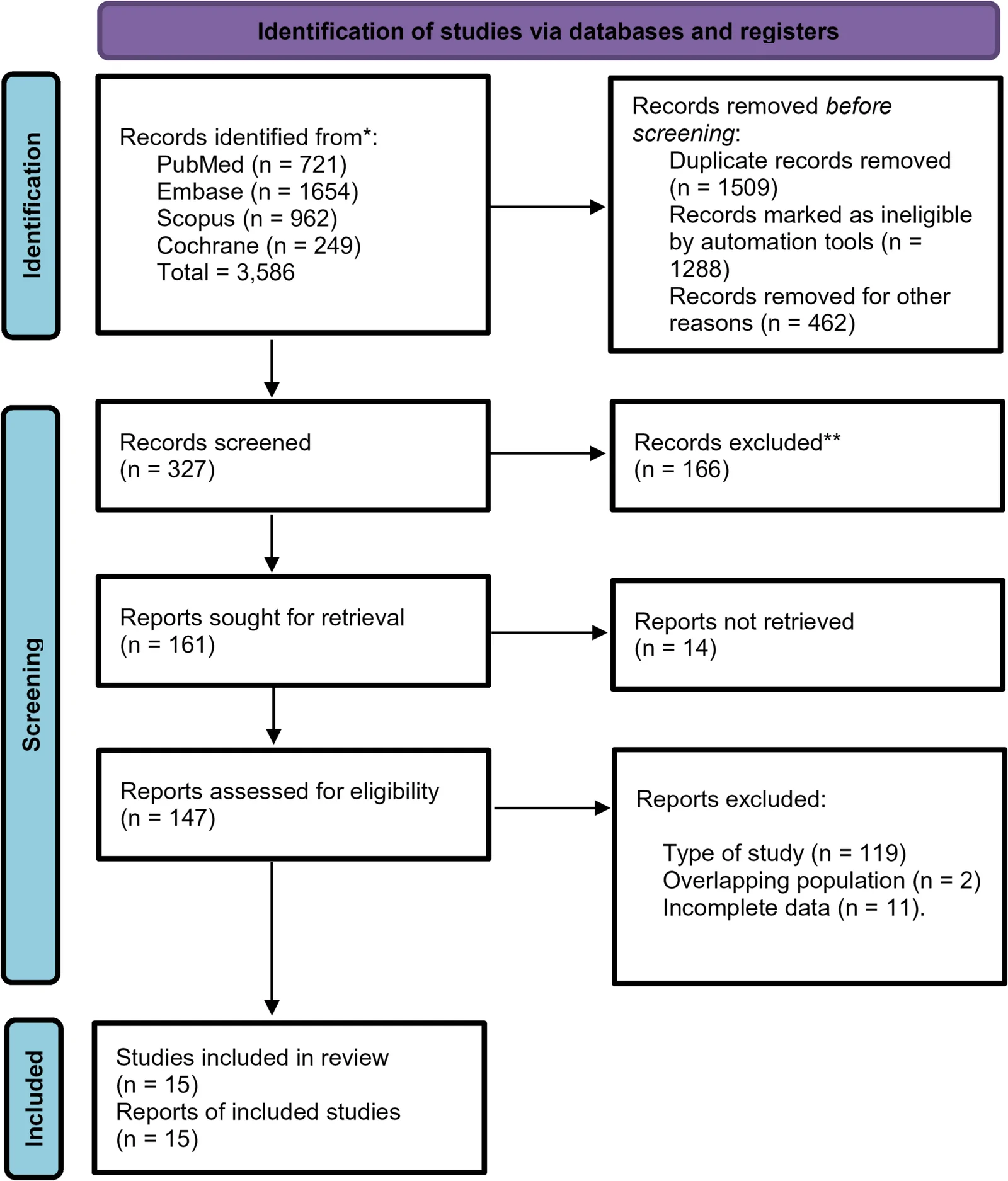
PRISMA diagram

The systematic search yielded 3,586 results: 721 from PubMed, 1654 from Embase, 962 from Scopus, and 249 from the Cochrane Library. Before the screening process, 1509 duplicate papers detected by software were manually verified for similarity and removed. A total of 1288 articles marked as ineligible by automation tools were removed, and 462 articles were removed for other reasons. A total of 161 reports were sought for retrieval, of which 14 could not be retrieved. Consequently, 147 full-text articles were assessed for eligibility. Of these, 132 were excluded because of incorrect study design (*n* = 119), incomplete data (*n* = 11), or overlapping populations (*n* = 2). Ultimately, 15 studies were included in the final synthesis (Fig. 1) [31–45].

### Study characteristics

**Fig. 2:**
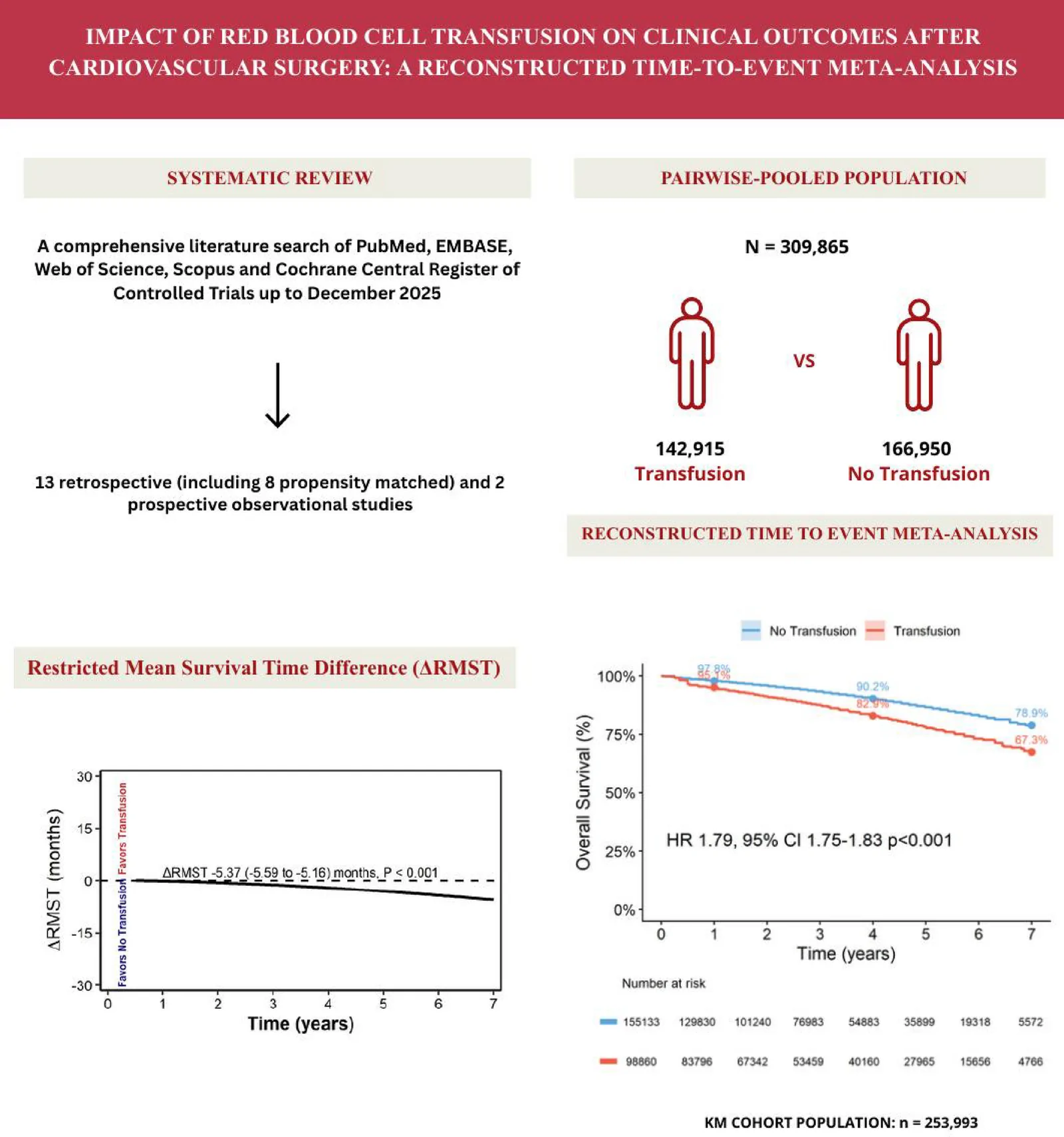
Central illustration

We included 15 studies (13 retrospective and 2 prospective), comprising 8 propensity score–matched analyses, 1 propensity score–based logistic regression without matching, and 6 studies without propensity score methods, comparing red blood cell transfusion with no transfusion in patients undergoing cardiovascular surgery. The characteristics of the included studies are presented in Table 1 and Supplementary S12. Of these, eight studies evaluated patients undergoing combined CABG with concomitant valve surgery, whereas six studies focused on isolated CABG procedures. Surgical approaches varied and included isolated on-pump CABG, isolated valvular surgery, aortic surgery, CABG with aortic or multivalve interventions, and mixed CABG ± valve populations, reflecting real-world clinical practice [31–45]. The number of patients, study design, type of cardiac surgery, mean age, number of females, body mass index (BMI), EuroSCORE, ejection fraction, hypertension (HTN), Dyslipidemia, Peripheral artery disease (PAD), and diabetes mellitus (DM) were collected from included studies and presented in Table 1. The mean age of the patients in the red blood cell transfusion group was approximately 69.8 years, and approximately 29.5% were female. In addition, 64.4% of the patients had hypertension, 65.2% had dyslipidemia, 16.0% had peripheral artery disease, 45.6% had coronary artery disease, and 27.9% had a history of diabetes mellitus. The median follow-up period ranged from 30 days to 9 years. Across the included studies, transfusion exposure was variably defined, most commonly as any perioperative red blood cell transfusion (Table 2). Several studies stratified exposure by dose, enabling prespecified subgroup analyses comparing high-dose (≥ 4 units) and low-dose (1–3 units) transfusions. Unless otherwise specified, patient counts reported for individual outcomes reflect only studies contributing data to that specific analysis. The reconstructed time-to-event analysis of long-term mortality included 253,993 patients (98,860 transfusion vs. 155,133 no transfusion) and 834,570 patient-years of follow-up, whereas the overall meta-analysis included a larger cumulative population of 309,865 patients (142,915 transfusion vs. 166,950 no transfusion) across all outcomes as shown in central illustration (Fig. 2).

**Table 1:** Baseline characteristics of included studies

Author year	No of patients: transfusion/No transfusion	Study Design	Country	Cardiac Surgery type	Age (years)* transfusion/ No transfusion	Female *n*(%) transfusion/ No transfusion	BMI (kg/m2)* transfusion/ No transfusion	EuroSCORE* transfusion/ No transfusion	Ejection fraction* transfusion/ No Transfusion	HTN *n*(%) transfusion/ No transfusion	DM *n*(%) Transfusion/ No transfusion	Follow-up
Bhaskar 2012^a^	3013/2329	Retrospective	Australia	CABG + VALVE	67 ± 9 / 62 ± 9	1,735 (57.6%) / 625 (19.8%)	27 ± 0.2 / 31 ± 0.2	10.4 / 4.3^a^		2,129 (70.7) / 716 (37.7)	750 (24.3) / 506 (21.7)	Up to 5 years
Santos 2013	1888/1116	Retrospective	Brazil	CABG	NA	NA	NA	NA	NA	NA	NA	1 year
Engoren 2009^a^	378/615	Retrospective	USA	VALVE	62 ± 14 / 60 ± 13	210 (55.6%) / 283 (46.0%)	NA	NA	51 ± 12 / 52 ± 11	254 (67) / 375 (61)	64 (17) / 76 (12)	Up to 5 years
	534/296			CABG + VALVE	70 ± 9 / 67 ± 10	220 (41%) / 110 (37%)	NA	NA	44 ± 14 / 48 ± 13	446 (84) / 224 (76)	193 (36%) / 74 (25%)	
Koster 2018^a^	2059 / 2059	Retrospective	Germany	CABG ± VALVE	65.3 ± 10.3 / 64.5 ± 10.1	367 (17.8%) / 305 (14.8%)	28.1 ± 4.5 / 28.3 ± 4.5	4.9 ± 5.7 / 4.4 ± 5.1	NA	NA	25.0 / 24.7	Median 4.05 years
Kuduvalli 2005^a^	940 / 2084	Retrospective	UK	CABG	66.3 ± 8.32 / 63.27 ± 8.46	307 (32.7%) / 296 (14.2%)	27.07 ± 3.93 / 28.07 ± 3.63	NA	NA	57.6 / 54.2	19.5 / 16.8	1 year
Ming 2019^a^	4937 / 3301	Retrospective	China	On-pump CABG ± VALVE	54.67 ± 12.61/ 49.67 ± 10.38	3051 (61.8%) / 1700 (51.5%)	22.62 ± 3.03 / 23.30 ± 3.02	NA	61 ± 9 / 61 ± 9	931 (18.9%) / 413 (12.5%)	412 (8.3%) / 119 (3.6%)	In-hospital only
Schwann 2023	116,766 / 141,632	Retrospective	USA	Isolated CABG (On-pump)	73.9 ± 6 / 71.9 ± 5.3	47,691 (40.8%) / 22,334 (15.8%)	28.6 ± 5.7 / 29.9 ± 5.4	NA	52 ± 13 / 54 ± 11	NA	55,588 (47.6%) / 61,635 (43.5%)	Long-term follow-up via CMS linkage
Stone 2012	570 / 921	Prospective	USA	Isolated CABG (On-pump)	NA	NA	NA	NA	NA	NA	NA	1 year
Sultan 2021^a^	4095 / 4095	Retrospective	USA	Open cardiac surgery (CABG ± VALVE; Multivalve)	68.33 ± 11.87/ 67.67 ± 11.12	1,563 (38.17%) / 1,509 (36.85%)	28.91 ± 5.83 / 28.92 ± 5.57	NA	53.33 ± 11.12/ 53.33 ± 11.12	342 (83.59) / 341 (83.49)	159 (39.02) / 157 (38.41)	Median 4.03 years
Sultan 2023^a^	224 / 224	Retrospective	USA	Aortic surgery	61.67 ± 14.18 / 60.17 ± 14.55	76 (33.9%) / 77 (34.4%)	NA	NA	56.67 ± 5.22 / 55.83 ± 7.46	NA	23 (10.3) / 19 (8.5)	5 years
Tantawy 2018^a^	446/446	Retrospective	USA	CABG (On-pump)	66.6 ± 10.9 / 66.5 ± 10.3	103 (23.2%) / 104 (23.2%)	30.0 ± 6.3 / 29.7 ± 5.8	NA	51.4 ± 13.9 / 51.2 ± 14.0	386 (86.6) / 381 (85.4)	183 (41.0) / 177 (39.7)	4 years
Tran 2020	1525 / 334	Prospective	Norway	CABG ± VALVE (Mixed)	73.22 ± 8.9 / 68.43 ± 9.75	522 (34.2%) / 23 (6.9%)	25.37 ± 3.78 / 26.53 ± 3.87	NA	NA	874 (57.3%) / 179 (53.6%)	344 (22.6%) / 72 (21.6%)	5 years
Vlot 2019^b^	488 / 2445	Retrospective	Norway	On-pump CABG ± AVR	73.67 ± 8.18 / 66.33 ± 9.64	239 (49.0%) / 419 (17.1%)	26.33 ± 3.72 / 27.33 ± 3.71	6 ± 2.97 / 3.33 ± 2.23	NA	321 (65.8) / 1466 (60.0)	166 (34.0) / 567 (23.2)	30-day
Bjursten 2016	5041/4866	Retrospective	Sweden	CABG + AVR	70.3 ± 9.8 / 65.3 ± 10.0	1843 (37%) / 702 (14%)	26.8 ± 4.9 / 27.6 ± 4.0	6.0 ± 3.3 / 3.9 ± 2.8	NA	NA	1110 (22%) / 1007 (21%)	1–12 years
Koçyiğit 2023	11/187	Retrospective	Turkey	Isolated CABG	69.70 ± 6.06 / 51.38 ± 9.96	8 (72.7%) / 20 (10.7%)	27.79 ± 4.08 / 28.76 ± 4.25	13.60 ± 18.27 / 5.71 ± 10.84	52.36 ± 9.83 / 55.76 ± 10.03	7 (63.6%) / 121 (64.7%)	6 (54.5%) / 80 (42.7%)	NA

BMI - Body Mass Index, HTN - Hypertension, DM - Diabetes mellitus, EuroSCORE - European System for Cardiac Operative Risk Evalua

tion, EuroSCORE ROD - European System for Cardiac Operative Risk Evaluation, Risk Of Death, SD - Standard Deviation

Data are presented as *Mean ± Standard Deviation and Percentage. All data is given for Transfusion or No Transfusion

^a^Propensity score-matching

^b^Propensity score–based logistic regression; no matching

**Table 2:** Definition of blood transfusion in included studies

Study	Blood transfusion definition
Bhaskar 2012^a^	Any perioperative blood transfusion during or ≤ 72 h after CABG
dos Santos 2013	Packed red blood cell transfusion (≥ 1 unit) Post-CABG
Engoren 2009^a^ (CABG+VALVE) Engoren 2009^a^ (CABG)	Any red blood cell transfusion
Koster 2017^a^	Red blood cell transfusion (1–2 units, leukocyte-depleted); Hb ≤ 8–10 g/dl (age-dependent)
Kuduvalli 2005^a^	Perioperative red blood cell transfusion during or ≤ 72 h after CABG
Ming 2019^a^	Any red blood cell transfusion/FFP/Platelet; unit-based analysis
Schwann 2023	Any red blood cell transfusion: Intra-and postoperative High dose (≥ 4 units) and Low dose (1–3 units)
Stone 2012	Any red blood transfusion post-CABG High dose (≥ 4 units) and Low dose (1–3 units)
Sultan 2021^a^	Packed red blood cell (pRBC) transfusion: Intra-and postoperative High dose (≥ 4 units) and Low dose (1–3 units)
Sultan 2023^a^	Any blood product (pRBC ± FFP/platelets/Cryoprecipitate) transfusion
Tantawy 2018^a^	Packed red blood cell (pRBC) transfusion (1–5 units)
Tran 2020	Red blood cell transfusion (≥ 1 unit)
Vlot 2019^b^	Red blood cell transfusion (≥ 1 unit)
Koçyiğit 2023	Red blood cell transfusion
Bjursten 2016	Leukocyte-depleted RBCs and Non-leukocyte-depleted RBCs High dose (≥ 4 units) and Low dose (1–3 units)

^a^Propensity score-matching, ^b^Propensity score–based logistic regression; no matching, pRBC: Packed red blood cells, CABG: Coronary artery bypass graft, FFP: Fresh frozen plasma

### Primary endpoints

#### 30-day mortality

**Fig. 3:**
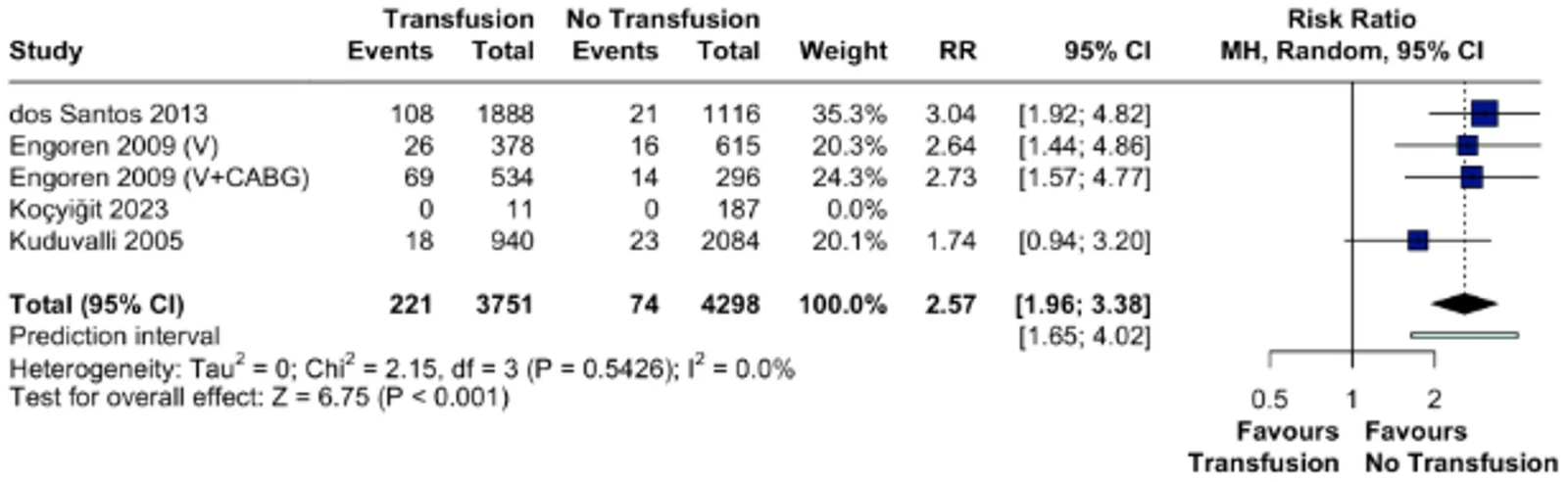
30-day mortality

Four studies reported 30-day mortality, with 221 of 3,751 patients (5.89%) in the red blood cell transfusion group and 74 of 4,298 patients (1.72%) in the no-transfusion group experiencing events. Red blood cell transfusion was associated with a significantly higher risk of 30-day mortality (RR = 2.57, 95% CI [1.96, 3.38], I² = 0.0%, *p* < 0.001; Fig. 3), which was supported by a prediction interval of 1.65–4.04. Sensitivity analysis showed that exclusion of dos Santos et al., 2013, Kuduvalli et al., 2005, and Koçyiğit et al., 2023 did not materially alter the pooled estimates and reduced heterogeneity to 0% (Supplementary S9a).

#### Overall Long-term survival analysis

**Fig. 4:**
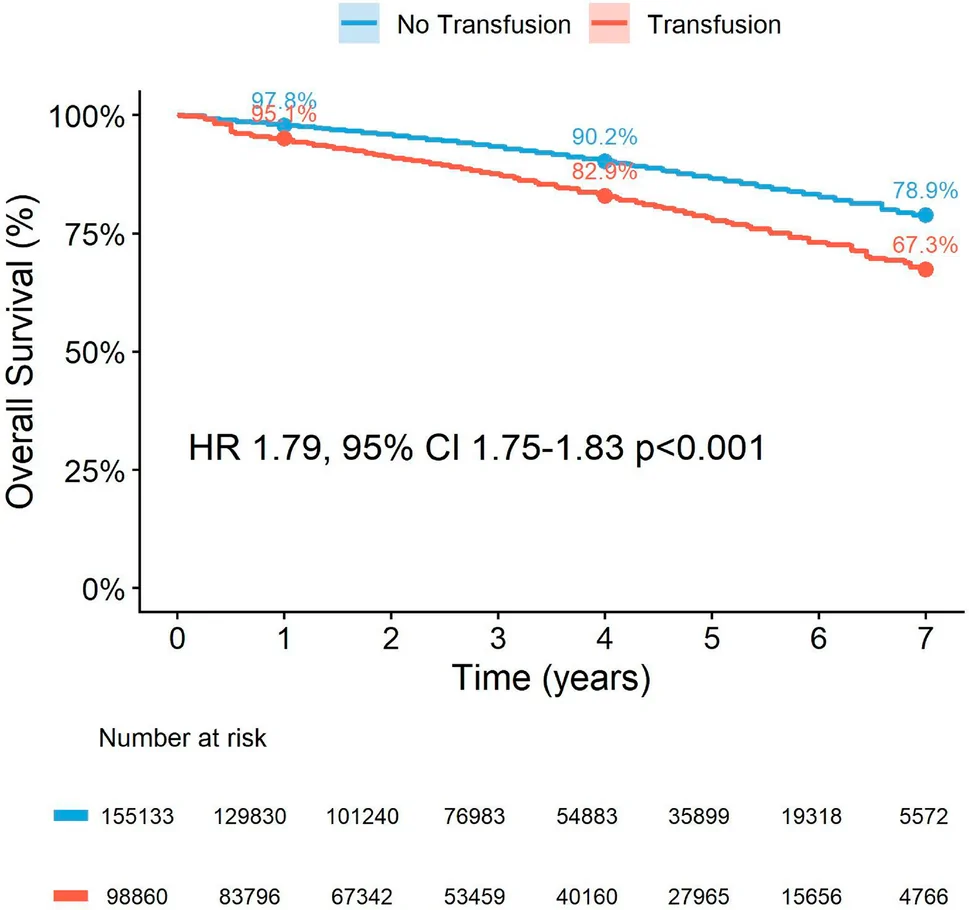
Kaplan-Meier plot analysis for overall long-term survival

**Fig. 5:**
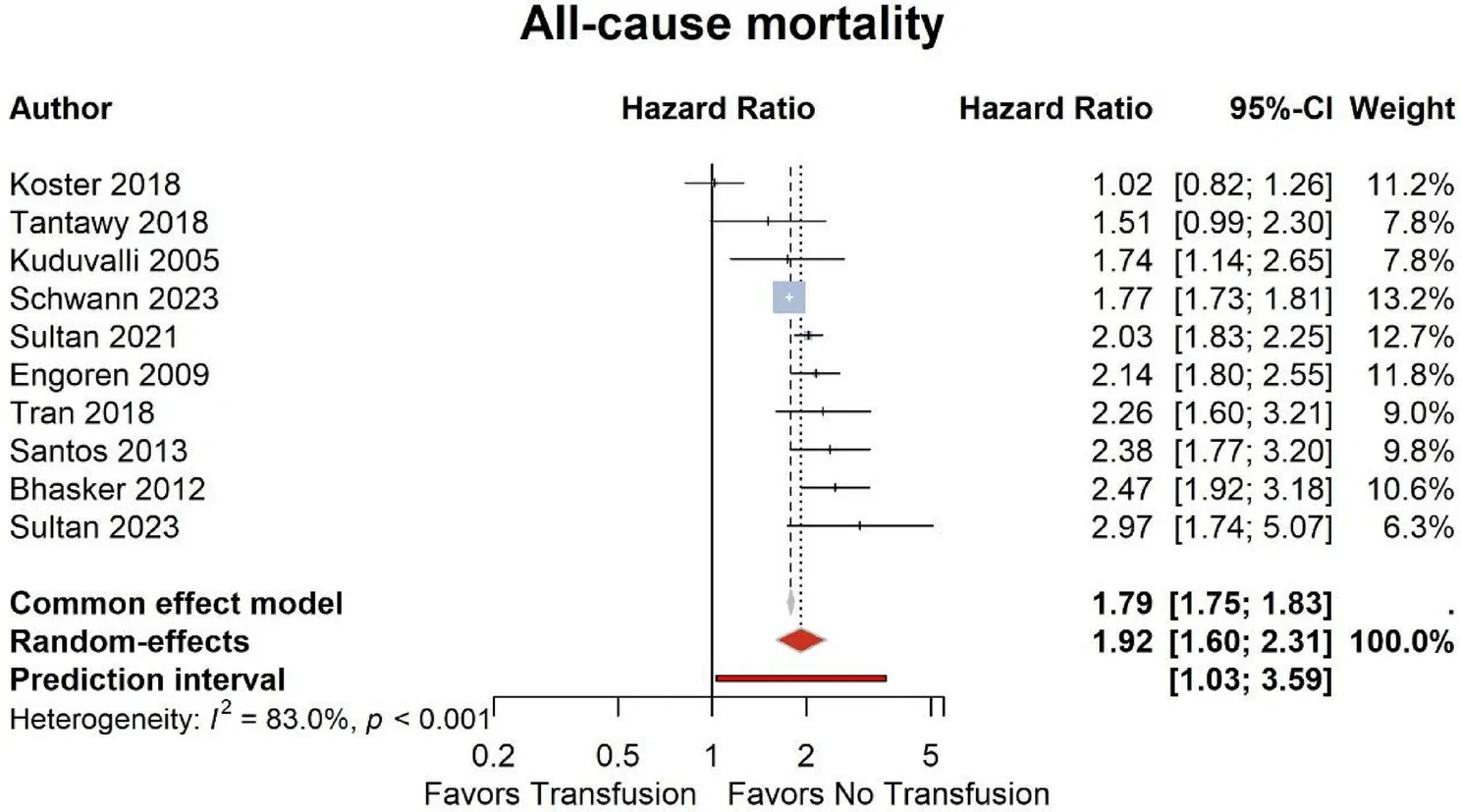
Forest plot of overall long-term all-cause mortality

Ten studies, including 253,993 patients (blood transfusion: 98,860 patients, 38.9%, and no blood transfusion: 155,133 patients, 61.1%), reported data on all-cause mortality during follow-up, spanning a total of 834,570 patient-years. At 1-year, the survival rates were 95.1% and 97.8% in patients with and without transfusion, respectively. At 7 years of follow-up, the survival rates were 67.3% and 78.9% in patients with and without transfusion, respectively (HR 1.79 [95% CI 1.75, 1.83]; *p* < 0.001; Fig. 4), with clear separation of Kaplan–Meier survival curves throughout follow-up, indicating worse long-term survival in the transfusion group. Overall long-term all-cause mortality was significantly higher among patients receiving red blood transfusions compared with those without transfusions (HR 1.92, 95% CI [1.60, 2.31], Fig. 5). For long-term mortality, the pooled hazard ratio was associated with substantial heterogeneity (I² = 92.6%). The corresponding prediction interval ranged from 1.03 to 3.59, indicating that although the magnitude of the association may vary across clinical settings, the direction of the effect remained consistent, with transfusion associated with increased mortality.

**Fig. 6:**
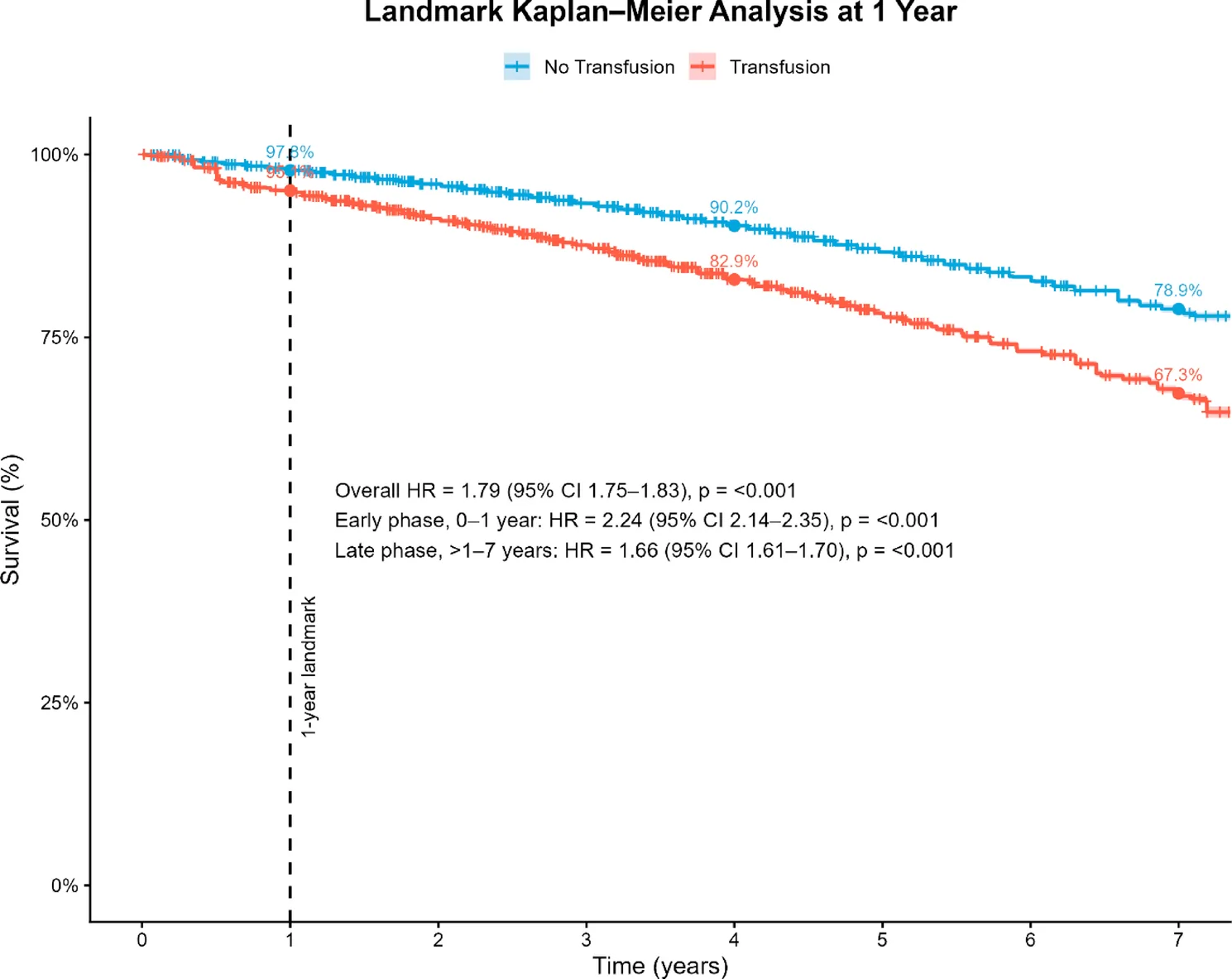
1-year landmark analysis for overall long-term survival

The proportional hazards assumption was violated (Grambsch–Therneau test *p* = 0.0003; Supplementary S3b), suggesting time-varying effects of transfusion on mortality. Time-varying hazard ratio analysis demonstrated that transfusion was associated with a significantly increased risk of mortality throughout follow-up. The hazard ratio was highest early after the procedure (HR ~ 3.0) and gradually declined over time, reaching approximately 1.4 by 7 years. Nevertheless, the hazard ratio remained consistently above unity, and the 95% confidence intervals did not cross the null value, indicating a persistent adverse association between transfusion and long-term mortality (Supplementary S3c). Landmark analysis at 1 year demonstrated a pronounced early mortality hazard associated with transfusion (0–1 year: HR 2.24, 95% CI [2.14, 2.35], *p* < 0.001), which decreased but remained significant during long-term follow-up (> 1–7 years: HR 1.66, 95% CI [1.61, 1.70], *p* < 0.001). The overall pooled hazard ratio for all-cause mortality was 1.79, 95% CI [1.75, 1.83], *p* < 0.001, indicating a persistent adverse association of transfusion with mortality over time (Fig. 6). The stratified log-rank test demonstrated a significant difference in long-term survival between the transfusion and no-transfusion groups after accounting for between-study variability (χ² = 2574, df = 1, *p* < 0.001), with poorer survival observed among patients who received transfusion. Restricted mean survival time analysis demonstrated significantly reduced survival among transfused patients at 7 years (ΔRMST − 5.37 months; 95% CI − 5.59 to -5.16 months; *p* < 0.001; Supplementary S3d), supporting a clinically meaningful adverse association with transfusion.

Median survival follow-up duration was 3.13 years (IQR 1.49–5.05), with 2.84 years (IQR 1.43–4.82) in the no-transfusion group and 3.28 years (IQR 1.49–5.23) in the transfusion group, although survival probabilities declined more rapidly over time among transfused patients (Supplementary S3a). Visual inspection of the funnel plot did not suggest significant asymmetry. Formal assessments using Begg’s rank correlation test and Egger’s regression test showed no evidence of publication bias (Begg’s *p* = 0.53; Egger’s *p* = 0.43; Supplementary S3e).

Leave-one-out sensitivity analyses confirmed the robustness of the findings. Exclusion of individual studies did not materially alter the pooled estimate for long-term mortality, with hazard ratios remaining statistically significant across all iterations (HR range: 1.87–2.04). The highest pooled estimate was observed after exclusion of Schwann et al. (HR 1.95, 95% CI 1.58–2.41), indicating that the overall effect was not driven by any single study. Although heterogeneity decreased substantially following exclusion of Koster et al. 2018 (I² = 64.6%), the pooled effect estimate remained largely unchanged, suggesting that this study contributed to between-study variability but did not influence the overall association (Supplementary S3f).

### Secondary endpoints

#### Atrial fibrillation

Across three studies, atrial fibrillation occurred in 1,409 of 5,622 patients (25.1%) in the transfusion group and in 964 of 5,974 patients in the no-transfusion group. Transfusion was associated with a significantly increased risk of atrial fibrillation (RR = 1.57, 95% CI [1.21, 2.04]; I² = 18.1%, *p* < 0.001; Supplementary S4c), with a prediction interval of 0.66–3.74. Sensitivity analysis demonstrated that heterogeneity was primarily driven by the studies by Stone et al., 2012, and Bjursten et al., 2016, as exclusion of these studies reduced the I² statistic to 0% (Supplementary S9e).

#### Acute kidney injury

Pooled analysis of three studies assessing acute kidney injury (AKI) demonstrated a significantly higher incidence in patients who received transfusion (359/2319; 15.48%) compared with those who did not (33/1479; 2.23%) (RR = 4.52, 95% CI [2.32, 8.80]; I² = 58.2%, *p* < 0.001; Supplementary S4d). This finding was supported by a wide prediction interval (0.40–50.76). Subgroup analysis showed no significant interaction between study design (propensity score–matched vs. non–propensity score–matched) and the effect of transfusion on AKI risk (*p* for interaction = 0.1132). Sensitivity analysis identified the study by Tran et al., 2020 as the primary source of heterogeneity, with exclusion reducing the I² statistic to 0% (Supplementary S9f).

#### Myocardial infarction

Five studies reported myocardial infarction outcomes. The pooled analysis demonstrated a significantly higher incidence of myocardial infarction among patients who received red blood cell transfusion (332/11,089; 2.99%) compared with those who did not receive transfusion (176/10,534; 1.67%) (RR = 2.32, 95% CI [1.60, 3.37], I² = 71.7%, *p* < 0.001; Supplementary S4e). This finding was supported by a prediction interval of 0.78–6.87. Sensitivity analysis showed that exclusion of the study by Stone et al., 2012 modestly reduced heterogeneity (I² = 35.6%), indicating that this study contributed to between-study variability (Supplementary S9g).

#### Pneumonia

Pneumonia was reported in five studies, and pooled analysis demonstrated a significantly higher risk among patients receiving red blood cell transfusion (1,031/12,839; 8.03%) compared with those who did not receive transfusion (192/10,870; 1.77%) (RR = 5.10, 95% CI [3.04, 8.57]; I² = 87.1%, *p* < 0.001; Supplementary S4f). The robustness of this finding was supported by a prediction interval of 1.11–23.51. Sensitivity analysis showed that exclusion of the study by Ming et al., 2019 modestly reduced heterogeneity (I² = 34.9%), indicating that this study contributed to the observed between-study variability (Supplementary S9h).

#### Reoperation for bleeding

Five studies reported reoperation for bleeding, with events occurring in 1,025 of 9,941 patients (10.31%) in the transfusion group and 86 of 10,293 patients (0.84%) in the no-transfusion group. Pooled analysis demonstrated that red blood cell transfusion was associated with a significantly higher risk of reoperation for bleeding compared with no transfusion (RR = 12.61, 95% CI [7.99, 19.90]; I² = 33.6%, *p* < 0.001; Supplementary S4g). This finding was supported by a prediction interval of 4.15–38.36. Sensitivity analysis showed that exclusion of the study by Bjursten et al., 2016 reduced heterogeneity to 0% (Supplementary S9i).

#### Sepsis

Four studies reported sepsis, with 240 of 12,269 patients (1.96%) in the transfusion group and 34 of 9,949 patients (0.34%) in the no-transfusion group experiencing events. The pooled analysis demonstrated a significantly higher risk of sepsis associated with red blood cell transfusion (RR = 7.49, 95% CI [2.64, 21.26]; I² = 80.2%, *p* < 0.001; Supplementary S4h). This finding was supported by a wide prediction interval (0.33–170.83), reflecting substantial between-study variability. Sensitivity analysis indicated that the heterogeneity was largely driven by the study by Ming et al., 2019, as exclusion of this study reduced the I² statistic to 0% (Supplementary S9j).

#### Sternal infection

Four studies reported sternal infection events in 62 of 8,272 patients (0.75%) in the transfusion group and 18 of 8,732 patients (0.21%) in the no-transfusion group. Pooled analysis demonstrated a significantly higher risk of sternal infection associated with red blood cell transfusion (RR = 3.97, 95% CI [2.31, 6.83]; I² = 0.0%, *p* < 0.001; Supplementary S4i). Sensitivity analysis showed that exclusion of Tantawy et al., 2018 did not materially alter the findings and yielded an I² of 0% (Supplementary S9k).

#### Stroke

Across six studies, a total of 321 stroke events occurred among 13,883 patients (2.31%) in the transfusion group, compared with 141 events among 14,694 patients (0.96%) in the no-transfusion group. Red blood cell transfusion was associated with a significantly higher risk of stroke relative to no transfusion (RR = 2.54, 95% CI [2.04, 3.16]; I² = 3.7%, *p* < 0.001; Supplementary S4j). Sensitivity analysis demonstrated minimal between-study heterogeneity, which was completely resolved after exclusion of the studies by Kuduvalli et al. (2005) and Sultan et al. (2021, 2023) (I² = 0%; Supplementary S9l).

### Subgroup analysis

#### Cardiac surgery type

##### 30-day mortality

In subgroup analyses stratified by surgery type, red blood cell transfusion was consistently associated with increased 30-day mortality following isolated CABG (RR 2.38), isolated valve surgery (RR 2.64), and mixed CABG + valve procedures (RR 2.73). There was no significant interaction by surgery type (*p* = 0.9), indicating that the association between transfusion and mortality did not differ across surgical categories (Supplementary S5a).

##### Long-term mortality

In subgroup analyses stratified by surgery type, red blood cell transfusion was associated with increased long-term mortality following aortic surgery (RR 2.80). In isolated CABG, transfusion demonstrated a non-significant trend toward increased mortality with substantial heterogeneity. There was no evidence of effect modification by surgery type (*p* = 0.64) (Supplementary S5c).

#### Study design

##### 30-day mortality

In subgroup analyses stratified by study design, red blood cell transfusion was associated with increased 30-day mortality in both non-propensity score-matched studies (RR 3.04) and propensity score-matched studies (RR 2.35). There was no significant interaction by study type (*p* = 0.38), indicating a consistent association between transfusion and 30-day mortality regardless of adjustment strategy (Supplementary S6a).

#### Transfusion dose

##### Long-term mortality

In dose-stratified subgroup analyses, high-dose transfusion (≥ 4 units) was associated with a significantly increased risk of long-term mortality (HR 2.27), whereas low-dose transfusion (1–3 units) was not. A significant interaction by transfusion dose was observed (*p* = 0.008), indicating a dose-dependent relationship between transfusion exposure and long-term mortality (Supplementary S7a).

#### Transfusion timing

##### Long-term mortality

In subgroup analyses based on transfusion timing, intraoperative red blood cell transfusion was associated with a significantly increased risk of long-term mortality (HR = 1.26, 95% CI [1.08, 1.47]; I² = 35%; *p* = 0.004; Supplementary S7b). In contrast, postoperative transfusion alone did not demonstrate a statistically significant association with long-term mortality (HR = 1.68, 95% CI [0.94, 2.99]; I² = 98%; *p* = 0.08; Supplementary S7c), with substantial between-study heterogeneity.

When both intraoperative and postoperative transfusions were combined, a significant increase in long-term mortality was observed (HR = 1.49, 95% CI [1.43, 1.55]; I² = 97%; *p* < 0.00001; Supplementary S7d), although this estimate was accompanied by considerable heterogeneity. The substantial heterogeneity in postoperative and combined intra- and postoperative long-term mortality analyses likely reflects differences in transfusion timing definitions, residual confounding by indication, variable patient risk profiles and adjustment strategies, and limited study numbers.

### Meta-regression

In meta-regression analyses of long-term mortality, no significant associations were observed with the proportion of patients with diabetes mellitus (β = 0.0011, SE 0.0092, 95% CI − 0.0223 to 0.0201; *p* = 0.907; Supplementary S10a), prior myocardial infarction (β = 0.0025, SE 0.0081, 95% CI − 0.0173 to 0.0222; *p* = 0.770; Supplementary S10b), peripheral artery disease (β = 0.0450, SE 0.0216, 95% CI − 0.0077 to 0.0978; *p* = 0.082; Supplementary S10c), urgent or emergent surgery (β = 0.3365, SE 0.1124, 95% CI − 1.0914 to 1.7644; *p* = 0.205;Supplementary S10e), age (β = 0.0076, SE 0.0191, 95% CI − 0.0567 to 0.0414; *p* = 0.707; Supplementary S10d), or female sex (β = 0.0049, SE 0.0097, 95% CI − 0.0170 to 0.0269; *p* = 0.624; Supplementary S10f) (Table 3).

**Table 3:** Meta regression of long-term mortality outcome

Covariate	β (SE)	95% CI	Unit of Increase	*p* value	Interpretation
Diabetes mellitus (%)	-0.0011(0.0092)	-0.0223 to 0.0201	Per 1% increase	0.907	No association
Myocardial infarction (%)	0.0025(0.0081)	-0.0173 to 0.0222	Per 1% Increase	0.770	No association
Peripheral artery diseases (%)	0.0450(0.0216)	-0.0077 to 0.0978	Per 1% Increase	0.082	No significant (borderline)
Urgent/emergent surgery (%)	0.3365(0.1124)	-1.0914 to 1.7644	Per 1% Increase	0.205	Not significant (very imprecise)
Age (years)	-0.0076(0.0191)	-0.0567 to 0.0414	Per 1-year Increase	0.707	No association
Female sex (%)	0.0049(0.0097)	-0.0170 to 0.0269	Per 1% Increase	0.624	No association

CI: Confidence interval, *β*: Regression coefficient, SE: Standard error, *p-value*: Probability value

### GRADE

Using the GRADE framework, the certainty of evidence varied from low to high across outcomes, largely reflecting the inclusion of non-randomized studies and consequent downgrading for risk of bias. Nonetheless, several outcomes—including 30-day mortality, stroke, acute kidney injury, and reoperation for bleeding—achieved moderate to high certainty, driven by large or very large effect sizes that justified upgrading despite the observational nature of the evidence. In contrast, heterogeneity and inconsistency lowered certainty for long-term mortality and myocardial infarction; however, the overall consistency and magnitude of effects across most outcomes support moderate confidence in the association between red blood cell transfusion and adverse cardiovascular outcomes. A summary of the GRADE is presented in Table 4.

**Table 4:** Assessment of the quality of evidence using the GRADE approach

Certainty assessment							Summary of findings				
Participants (studies) Follow-up	Risk of bias	Inconsistency	Indirectness	Imprecision	Publication bias	Overall certainty of evidence	Study event rates (%)		Relative effect (95% CI)	Anticipated absolute effects	
							With No Transfusion	With Transfusion		Risk with No Transfusion	Risk difference with Transfusion
**30-day mortality**											
8049 (5 non-randomised studies)	not serious^a^	not serious	not serious	not serious	strong association^b^	⨁⨁⨁◯ Moderate^a, b^	74/4298 (1.7%)	221/3751 (5.9%)	**RR 2.57** (1.96 to 3.38)	74/4298 (1.7%)	**27 more per 1**,**000** (from 17 more to 41 more)
**Long term mortality**											
4348 (4 non-randomised studies)	not serious^a^	serious^c^	not serious	not serious	strong association^b^	⨁⨁◯◯ Low^a, b,c^	267/1788 (14.9%)	461/2560 (18.0%)	**RR 2.39** (1.42 to 4.03)	267/1788 (14.9%)	**208 more per 1**,**000** (from 63 more to 452 more)
**Atrial Fibrillation**											
11,596 (3 non-randomised studies)	not serious^a^	not serious	not serious	not serious	none	⨁⨁◯◯ Low^a^	964/5974 (16.1%)	1409/5622 (25.1%)	**RR 1.57** (1.21 to 2.04)	964/5974 (16.1%)	**92 more per 1**,**000** (from 34 more to 168 more)
**Myocardial Infarction**											
21,623 (5 non-randomised studies)	not serious^a^	serious^c^	not serious	not serious	strong association^b^	⨁⨁◯◯ Low^a, b,c^	176/10,534 (1.7%)	332/11,089 (3.0%)	**RR 2.32** (1.60 to 3.37)	176/10,534 (1.7%)	**22 more per 1**,**000** (from 10 more to 40 more)
**Stroke**											
28,577 (6 non-randomised studies)	not serious^a^	not serious	not serious	not serious	strong association^b^	⨁⨁⨁◯ Moderate^a, b^	141/14,694 (1.0%)	321/13,883 (2.3%)	**RR 2.54** (2.04 to 3.16)	141/14,694 (1.0%)	**15 more per 1**,**000** (from 10 more to 21 more)
**Acute Kidney Injury**											
3798 (3 non-randomised studies)	not serious^a^	not serious	not serious	not serious	strong association^b^	⨁⨁⨁◯ Moderate^a, b^	33/1479 (2.2%)	359/2319 (15.5%)	**RR 4.52** (2.32 to 8.80)	33/1479 (2.2%)	**79 more per 1**,**000** (from 29 more to 174 more)
**Reoperation for Bleeding**											
20,234 (5 non-randomised studies)	not serious^a^	not serious	not serious	not serious	very strong association^b^	⨁⨁⨁⨁ High^a, b^	86/10,293 (0.8%)	1025/9941 (10.3%)	**RR 12.61** (7.99 to 19.90)	86/10,293 (0.8%)	**97 more per 1**,**000** (from 58 more to 158 more)

Red blood cell transfusion compared to no transfusion after Cardiovascular surgery  Bibliography:

CI: confidence interval; RR: risk ratio

Explanations

a. Studies were not randomised

b. Upgraded due to large magnitude of effect

c. Statistical analyses showed a greater heterogeneity

### Publication bias

Egger’s regression test was performed to assess the presence of small-study effects and potential publication bias for each outcome. Across all evaluated outcomes—including long-term mortality, 30-day mortality, reoperation for bleeding, sternal infection, atrial fibrillation, pneumonia, stroke, myocardial infarction, acute kidney injury, and sepsis—the intercept estimates were not statistically significant (all *p* values > 0.05). These findings indicate no evidence of funnel plot asymmetry for any of the outcomes analyzed (Supplementary S8).

### Risk of bias assessment

In the ROBINS-I assessment, 12 studies were rated as having moderate risk of bias and 3 as having serious risk of bias. All studies with concerns exhibited bias in the confounding domain. Serious risk of bias was predominantly attributable to residual confounding, with additional concerns related to participant selection and outcome measurement in select studies, while other domains were largely judged to be at low risk (Supplementary S11).

### Discussion

In this meta-analysis of adult cardiovascular surgery patients, perioperative RBC transfusion was consistently associated with increased 30-day and long-term mortality, as well as higher rates of cardiac (MI, stroke, AF) and non-cardiac complications (AKI, pneumonia, sepsis). These associations persisted across surgery types, study designs, and adjustment strategies, but there was considerable between-study heterogeneity. The corresponding 95% prediction intervals for most outcomes except long-term all-cause mortality, spanned the null, underscoring that the excess risk may not be realized uniformly in all settings. Importantly, given the observational design of the data, our findings reflect association rather than proven causation.

Our findings are consistent with and extend prior observational evidence linking RBC transfusion to adverse outcomes following cardiovascular surgery [46–49]. However, previous meta-analyses in different populations have yielded mixed conclusions. In a meta-analysis of ICU patients, any apparent mortality excess with transfusion disappeared after adequate adjustment (pooled adjusted RR ~ 1.0) [50]. In contrast, our focus on cardiovascular surgery, a field with high baseline bleeding risk and comorbidity, may have amplified detectable associations. It is also notable that randomized trials of transfusion strategies (predominantly in acute coronary syndrome or critical illness) have generally shown no mortality benefit from liberal transfusion [51, 52]. A recent Cochrane review reported essentially identical 30-day mortality (RR ≈ 0.99; 95% CI 0.86–1.15) and stroke rates (RR ≈ 0.84; 95% CI 0.64–1.09) comparing liberal vs. restrictive hemoglobin thresholds [53]. This discrepancy likely reflects confounding by indication in observational data- sicker surgical patients both bleed more and receive more transfusions. To our knowledge, the present analysis is the first meta-analysis to pool both overall and dose-stratified data specifically on RBC transfusion and cardiovascular surgical outcomes, offering a more comprehensive synthesis than prior work.

While the association between transfusion and 30-day mortality was consistent and statistically robust, long-term mortality estimates demonstrated substantial heterogeneity and wide prediction intervals, indicating variability in effect magnitude across clinical settings. An important consideration when interpreting these findings is the substantial heterogeneity observed across several outcomes. Long-term mortality demonstrated considerable between-study heterogeneity (I² > 90%), and substantial heterogeneity was also observed for myocardial infarction, pneumonia, and sepsis. Although subgroup analyses, leave-one-out sensitivity analyses, and meta-regression were performed, much of the heterogeneity remained unexplained. Furthermore, several prediction intervals crossed the line of no effect, indicating that the observed associations may not be consistently reproduced across all patient populations, surgical settings, and healthcare system Therefore, the pooled estimates should be viewed as average associations across diverse clinical contexts rather than universally applicable effect estimates. The association between transfusion and reoperation for bleeding should be interpreted with particular caution, as perioperative bleeding frequently precedes both transfusion and reoperation, making this outcome susceptible to reverse causation and confounding by indication. Stone et al. 2012 was derived from the ACUITY trial and included patients with acute coronary syndromes who underwent CABG during the index hospitalization; therefore, its population differs from routine elective cardiac surgery cohorts and may contribute to clinical heterogeneity.

Time-to-event analyses indicate an association between perioperative blood transfusion and reduced survival; however, time-varying hazards and potential confounding warrant cautious interpretation [54]. Although the pooled hazard ratio and reconstructed Kaplan–Meier analyses yielded slightly different numerical estimates, both methods consistently demonstrated higher long-term mortality among transfused patients. Such differences are expected because aggregate-data hazard ratio meta-analysis and reconstructed individual patient data analyses apply different weighting schemes and handle censoring differently. Importantly, dose-stratified analyses demonstrated a significant association between high-volume transfusion and long-term mortality, whereas low-dose transfusion was not associated with excess risk. This finding may reflect a dose–response relationship; however, it may also indicate that transfusion volume serves as a surrogate for the severity of perioperative bleeding and physiological insult [55]. Overall, the considerable heterogeneity and subgroup findings caution against overgeneralizing the pooled point estimates to every clinical scenario.

Although most studies demonstrated worse survival among transfused patients, Koster et al. reported no significant association with mortality between groups. This discrepancy may reflect the exclusive evaluation of small-volume leukocyte-depleted transfusions and rigorous propensity matching, which may attenuate inflammatory effects and reduce confounding by indication. Importantly, patients who receive RBC transfusions after cardiovascular surgery often represent a clinically higher-risk population. Several studies reported that transfused patients were older, had higher operative risk scores, lower baseline hemoglobin levels, greater perioperative bleeding, longer cardiopulmonary bypass and aortic cross-clamp times, and more complex surgical procedures. These factors are themselves independently associated with adverse postoperative outcomes and may not have been fully captured by multivariable adjustment or propensity score methodologies. Therefore, the observed associations between transfusion and adverse outcomes may partly reflect residual confounding by indication rather than a direct biological effect of transfusion itself. Conversely, Sultan et al. reported a stronger association favoring no transfusion, likely related to the higher-risk aortic surgical population studied, where transfusion may serve as a marker of operative complexity and perioperative instability. These differences likely contributed to the substantial heterogeneity observed (I² = 83%), which decreased to 69% after exclusion of Koster et al., highlighting transfusion dose, surgical complexity, and patient selection as important sources of variability.

Similarly, timing-stratified analyses showed that intraoperative transfusion was associated with increased long-term mortality, while postoperative transfusion alone was not statistically significant (*p* = 0.08) and demonstrated extreme heterogeneity; combined intra- and postoperative exposure was associated with increased long-term mortality (HR 1.49) with considerable heterogeneity.

Collectively, these timing findings may indicate that early or sustained transfusion exposure potentially contributes to physiologic stress such as ongoing bleeding, prolonged bypass time, hemodilution, inflammatory activation, or hemodynamic instability, though residual confounding remains a highly plausible explanation, particularly for postoperative transfusion, where definitions and patient selection varied substantially.

Meta-regression did not identify clear effect modification by baseline covariates (like diabetes, prior MI, PAD, urgency, age, and female sex), implying that measured study-level clinical differences did not explain the heterogeneity observed in long-term mortality. The meta-regression analyses should be interpreted as exploratory because the limited number of included studies reduced the ability to reliably detect sources of between-study heterogeneity.

There are plausible pathophysiological reasons why transfusions might influence cardiovascular outcomes. Allogeneic RBC units can trigger immune and inflammatory responses. Furthermore, transfused blood may precipitate volume overload or transmit bioactive substances and cytokines, leading to endothelial activation [56]. The immuno-mediated reactions and changes in deformability of stored RBC could explain increased acute kidney injury and short-term mortality in cardiac patients receiving RBCs [57]. Transfusion-related immunomodulation (TRIM) and procoagulant microparticles from stored blood may also contribute to thrombosis and stroke [58]. These mechanisms lend credence to the associations we observed, but they do not exclude the alternative interpretation that transfusion simply identifies patients with more severe illness. The magnitude of several observed associations warrants caution. For example, the risks of reoperation for bleeding (RR 12.61), sepsis (RR 7.49), and pneumonia (RR 5.10) were substantially elevated among transfused patients. Such large effect sizes are unlikely to be explained solely by the biological effects of transfusion and more likely reflect, at least in part, confounding by indication. Patients who require transfusion frequently have more severe perioperative bleeding, prolonged operative times, greater hemodynamic instability, increased need for reintervention, and a higher burden of comorbid illness. Therefore, transfusion may function as a surrogate marker of perioperative severity rather than a direct cause of these complications. Although biologically plausible mechanisms linking transfusion to adverse outcomes exist, the observational nature of the available evidence precludes definitive separation of transfusion-related effects from the underlying clinical circumstances that prompted transfusion. In summary, while there is biological plausibility for harm, our data do not prove a direct causal pathway.

Given that all included studies were observational, the certainty of evidence ranged from low to high across outcomes. We used standard GRADE and ROBINS-I criteria to evaluate risk of bias, inconsistency, indirectness, imprecision, and publication bias. Several outcomes were downgraded because of confounding by indication, residual confounding, heterogeneity, and imprecision. Consequently, confidence in the estimated effects varies across outcomes and the findings should be interpreted cautiously.

Nonetheless, the pattern of associations provides important observational evidence regarding the relationship between transfusion exposure and postoperative outcomes. However, it is important to emphasize that this meta-analysis compared transfused and non-transfused patients rather than evaluating restrictive versus liberal transfusion strategies. Therefore, the findings should not be interpreted as evidence supporting any particular transfusion threshold. Randomized trials comparing restrictive and liberal transfusion strategies have generally reported no significant differences in mortality or major cardiovascular outcomes, suggesting that the associations observed in observational studies may be substantially influenced by confounding by indication and differences in baseline patient severity. Consequently, our results should be viewed as hypothesis-generating and complementary to, rather than a substitute for, evidence from randomized transfusion-strategy trials. Guidelines (with moderate-quality evidence) recommend a transfusion threshold of around 7–8 g/dL for hospitalized adults, including those undergoing cardiac surgery [59]. A Cochrane meta-analysis likewise found no survival or stroke benefit from more liberal transfusion protocols [53]. Taken together, the evidence supports judicious use of RBCs, reserving transfusion for true clinical necessity.

### Strengths

The strengths of this study are: first, it was a large, pooled population with long-term follow-up data. Second, a number of advanced methods were utilized: reconstructed individual patient survival analysis, RMST difference analysis, landmark analysis, Grambsch-Therneau test, time-varying hazard ratio analysis, frailty modeling, GRADE, meta-regression analysis and subgroup analysis. Third, most of the results were stable under sensitivity analyses with no important evidence of publication bias.

### Limitations

This study has several limitations. First, the evidence base was entirely observational, making the pooled estimates susceptible to confounding by indication and residual confounding. In the ROBINS-I assessment, confounding was the dominant source of bias; most studies were judged at moderate risk of bias, and some at serious risk of bias. Additionally, because the analysis compared transfused versus non-transfused patients rather than restrictive versus liberal transfusion strategies, the results cannot be used to determine optimal transfusion thresholds or guide transfusion decision-making directly.

Key determinants of both transfusion exposure and postoperative outcomes—including bleeding severity, nadir hemoglobin concentration, cardiopulmonary bypass duration, aortic cross-clamp time, surgical complexity, hemodynamic instability, and institutional transfusion practices—were inconsistently reported and adjusted for across studies. Consequently, residual confounding by indication cannot be excluded and may account for part of the observed associations between transfusion and adverse clinical outcomes. Furthermore, important perioperative variables such as bleeding severity, nadir hemoglobin, cardiopulmonary bypass duration, cross-clamp time, higher EuroSCORE values and operative complexity were not uniformly reported, limiting our ability to determine whether transfusion itself or the underlying severity of illness primarily accounted for the observed associations. Several clinically relevant blood-management variables, including cardiopulmonary bypass duration, transfusion protocols, antifibrinolytic use, cell saver utilization, and anticoagulation strategies, were inconsistently reported across studies and therefore could not be comprehensively analyzed. Similarly, nadir hemoglobin levels and perioperative blood loss were not consistently reported across studies, limiting our ability to evaluate their contribution to transfusion requirements and subsequent clinical outcomes.

Therefore, residual confounding and unmeasured sources of heterogeneity cannot be excluded.

Second, there was clinical and methodological heterogeneity across studies, including variability in surgical case mix and perioperative management, which likely contributed to between-study inconsistency. Although multiple exploratory analyses were undertaken, a substantial proportion of heterogeneity remained unexplained, limiting confidence in the generalizability of pooled effect estimates to individual clinical settings. For several outcomes, prediction intervals crossed the null, limiting the generalizability of a single pooled estimate to all settings.

Third, exposure definitions differed across studies (e.g., “any transfusion,” different perioperative windows, and variable handling of concomitant blood products), which may have increased misclassification and reduced comparability. This limitation is particularly relevant for timing analyses: postoperative-only and combined intra-/postoperative transfusion estimates were highly heterogeneous, reflecting differences in timing definitions and clinical context.

Fourth, publication bias and small-study effects cannot be excluded. Although Egger testing did not suggest asymmetry for assessed outcomes, these tests may be underpowered when few studies contribute to an endpoint.

Fifth, long-term mortality estimates were derived from Kaplan Meier-reconstructed individual patient data rather than original patient-level data; accordingly, results depend on the quality of published curves and accompanying risk tables and cannot recover covariate-adjusted effects or fully eliminate confounding by indication. Moreover, evidence of non-proportional hazards implies that the relative effect varies over follow-up; therefore, the pooled hazard ratio should be interpreted as a time-averaged estimate, and the accompanying restricted mean survival time difference provides a more robust absolute summary of long-term survival impact.

Furthermore, the certainty of evidence varied by outcome. In the GRADE assessment, certainty was moderate for 30-day mortality, stroke, and acute kidney injury; high for reoperation for bleeding; and low for long-term mortality, atrial fibrillation, and myocardial infarction, reflecting the nonrandomized design and, for some outcomes, inconsistency and imprecision.

This range in certainty supports stronger confidence in some associations (e.g., short-term mortality, stroke, AKI, bleeding/reoperation signals) than others (notably long-term mortality and MI), and it reinforces the need for randomized evidence that can more definitively separate transfusion effects from the underlying clinical severity that prompts transfusion.

An additional limitation is the variability in transfusion definitions across studies, ranging from any RBC transfusion to specific unit thresholds or broader blood product exposure definitions, which may have contributed to clinical heterogeneity and limited comparability across studies.

Most included studies did not report adjusted effect estimates in a consistent manner, and adjustment strategies varied considerably across studies. Consequently, pooled analyses were based primarily on unadjusted event data, which may increase susceptibility to residual confounding and confounding by indication. For long-term mortality, adjusted hazard ratios were pooled when available; however, adjusted estimates were not consistently reported for short-term and secondary outcomes.

Although the review focused on RBC transfusion, a small number of included studies incorporated broader transfusion definitions that included additional blood products. This variability in exposure definitions may have contributed to clinical heterogeneity and should be considered when interpreting the findings.

## Conclusion

In this meta-analysis of observational studies, perioperative RBC transfusion after cardiovascular surgery was associated with increased short- and long-term mortality and higher rates of several adverse postoperative complications. However, these findings should be interpreted with caution. Patients receiving transfusions frequently represent a higher-risk population with greater perioperative bleeding, more complex surgical procedures, longer operative times, lower hemoglobin levels, and greater physiological instability, all of which may contribute to the observed associations. Consequently, transfusion may act not only as a potential mediator of adverse outcomes but also as a marker of underlying clinical severity.

Furthermore, substantial heterogeneity was observed for several outcomes, particularly long-term mortality, and importantly, the prediction intervals did not cross the null value, suggesting that although the magnitude of the association may vary across different clinical settings, the direction of the effect remained consistent, with RBC transfusion being associated with increased mortality. Given the observational nature of the included studies, residual confounding and confounding by indication cannot be excluded, and definitive causal inferences cannot be made. Although Schwann et al. contributed the largest proportion of patients to the long-term mortality analysis, leave-one-out sensitivity analyses demonstrated consistent findings after its exclusion, supporting the robustness of the pooled estimate.

Importantly, this analysis compared transfused and non-transfused patients and did not evaluate restrictive versus liberal transfusion strategies. Therefore, these findings should not be interpreted as evidence supporting any specific transfusion threshold or strategy. Future adequately powered randomized trials in cardiovascular surgery populations are needed to determine whether the observed associations are directly attributable to transfusion itself or primarily reflect the underlying clinical circumstances that necessitate transfusion, and to establish optimal transfusion practices based on high-certainty evidence.

## Supplementary Information

Below is the link to the electronic supplementary material.

Supplementary Material 1.

## Data Availability

All data generated or analyzed during this study are included in this published article and its supplementary information files. Additional data may be available from the corresponding author upon reasonable request.

## Abbreviations

CABG: Coronary artery bypass graft
CI: Confidence interval
MD: Mean difference
RR: Risk ratio
GRADE: Grading of Recommendations, Assessment, Development, and Evaluation
TSA: Trial sequential analysis
PRISMA: Preferred Reporting Items for Systematic Reviews and Meta-Analysis
PROSPERO: Prospective Register of Systematic Reviews
AKI: Acute kidney injury
MI: Myocardial infarction
Afib: Atrial fibrillation
ROBINS-I: The Risk Of Bias In Non-randomized Studies of Interventions
PIs: Prediction intervals
MH: Mantel-Haenszel test
pRBC: Packed red blood cells
dRMST: Difference in Restricted Mean Survival Time
KM curves: Kaplan–Meier survival curves
