# Supplementary material for: Impact of red blood cell transfusion on clinical outcomes after cardiovascular surgery: a reconstructed time-to-event meta-analysis
Source: J Cardiothorac Surg. 2026 Jul 29;21:850. doi: 10.1186/s13019-026-04549-0 (PMC13712051; doi:10.1186/s13019-026-04549-0)
Supplement: Supplementary file 1 [file 13019_2026_4549_MOESM1_ESM.docx]

**Supplemental Material**

**Impact of Red Blood Cell Transfusion on Clinical Outcomes After Cardiovascular Surgery: A Reconstructed Time-to-Event Meta-Analysis**

**Supplemental Files Index**

**Supplementary S1 -** Preferred Reporting Items for Systematic Reviews and Meta-Analysis (PRISMA) checklist.

**Supplementary S2-** Research Question, PICOTT, and Search Strategy

**Supplementary S3-** Pooled Kaplan-Meier analysis for overall survival.

**Supplementary S4-** Forest plots for primary and secondary outcomes

**Supplementary S5**- Forest plots for subgroup analysis based on cardiac surgery type

**Supplementary S6**- Forest plots for subgroup analysis based on study design

**Supplementary S7**- Forest plots for subgroup analysis of long-term mortality based on transfusion timing and dose

**Supplementary S8-** Funnel plots and egger’s regression test for outcomes

**Supplementary S9-** Forest plots for leave one out sensitivity analysis

**Supplementary S10-** Bubble plots for long-term mortality (pooled hazard ratios)

**Supplementary S11-** Quality assessment using ROBINS - I

**Supplemental S12-** Propensity Score Matching (PSM) Protocol Summary Table

**Supplementary S1 -** Preferred Reporting Items for Systematic Reviews and Meta-Analysis (PRISMA) checklist

**PRISMA checklist**

| **Section and Topic** | **Item #** | **Checklist item** | **Page where item is reported** |
| --- | --- | --- | --- |
| **TITLE** | | |  |
| Title | 1 | Identify the report as a systematic review. | 1 |
| **ABSTRACT** | | |  |
| Abstract | 2 | See the PRISMA 2020 for Abstracts checklist. | 1 |
| **INTRODUCTION** | | |  |
| Rationale | 3 | Describe the rationale for the review in the context of existing knowledge. | 4,5 |
| Objectives | 4 | Provide an explicit statement of the objective(s) or question(s) the review addresses. | 4,5 |
| **METHODS** | | |  |
| Eligibility criteria | 5 | Specify the inclusion and exclusion criteria for the review and how studies were grouped for the syntheses. | 5,6 |
| Information sources | 6 | Specify all databases, registers, websites, organizations, reference lists and other sources searched or consulted to identify studies. Specify the date when each source was last searched or consulted. | 5,6 |
| Search strategy | 7 | Present the full search strategies for all databases, registers and websites, including any filters and limits used. | Supplementary S2 |
| Selection process | 8 | Specify the methods used to decide whether a study met the inclusion criteria of the review, including how many reviewers screened each record and each report retrieved, whether they worked independently, and if applicable, details of automation tools used in the process. | 6,7 |
| Data collection process | 9 | Specify the methods used to collect data from reports, including how many reviewers collected data from each report, whether they worked independently, any processes for obtaining or confirming data from study investigators, and if applicable, details of automation tools used in the process. | 6,7 |
| Data items | 10a | List and define all outcomes for which data were sought. Specify whether all results that were compatible with each outcome domain in each study were sought (e.g. for all measures, time points, analyses), and if not, the methods used to decide which results to collect. | 7 |
|  | 10b | List and define all other variables for which data were sought (e.g. participant and intervention characteristics, funding sources). Describe any assumptions made about any missing or unclear information. | 7 |
| Study risk of bias assessment | 11 | Specify the methods used to assess risk of bias in the included studies, including details of the tool(s) used, how many reviewers assessed each study and whether they worked independently, and if applicable, details of automation tools used in the process. | 8 |
| Effect measures | 12 | Specify for each outcome the effect measure(s) (e.g. risk ratio, mean difference) used in the synthesis or presentation of results. | 8,9 |
| Synthesis methods | 13a | Describe the processes used to decide which studies were eligible for each synthesis (e.g. tabulating the study intervention characteristics and comparing against the planned groups for each synthesis (item #5)). | 8,9 |
|  | 13b | Describe any methods required to prepare the data for presentation or synthesis, such as handling of missing summary statistics, or data conversions. | 8,9 |
|  | 13c | Describe any methods used to tabulate or visually display results of individual studies and syntheses. | 8,9 |
|  | 13d | Describe any methods used to synthesize results and provide a rationale for the choice(s). If meta-analysis was performed, describe the model(s), method(s) to identify the presence and extent of statistical heterogeneity, and software package(s) used. | 8,9 |
|  | 13e | Describe any methods used to explore possible causes of heterogeneity among study results (e.g. subgroup analysis, meta-regression). | 8,9 |
|  | 13f | Describe any sensitivity analyses conducted to assess robustness of the synthesized results. | 8,9 |
| Reporting bias assessment | 14 | Describe any methods used to assess risk of bias due to missing results in a synthesis (arising from reporting biases). | 8 |
| Certainty assessment | 15 | Describe any methods used to assess certainty (or confidence) in the body of evidence for an outcome. | 8 |
| **RESULTS** | | |  |
| Study selection | 16a | Describe the results of the search and selection process, from the number of records identified in the search to the number of studies included in the review, ideally using a flow diagram. | 10 |
|  | 16b | Cite studies that might appear to meet the inclusion criteria, but which were excluded, and explain why they were excluded. | 10 |
| Study characteristics | 17 | Cite each included study and present its characteristics. | 11,12 |
| Risk of bias in studies | 18 | Present assessments of risk of bias for each included study. | 35 |
| Results of individual studies | 19 | For all outcomes, present, for each study: (a) summary statistics for each group (where appropriate) and (b) an effect estimate and its precision (e.g. confidence/credible interval), ideally using structured tables or plots. | NA |
| Results of syntheses | 20a | For each synthesis, briefly summarise the characteristics and risk of bias among contributing studies. | 20-35 |
|  | 20b | Present results of all statistical syntheses conducted. If meta-analysis was done, present for each the summary estimate and its precision (e.g. confidence/credible interval) and measures of statistical heterogeneity. If comparing groups, describe the direction of the effect. | 20-35 |
|  | 20c | Present results of all investigations of possible causes of heterogeneity among study results. | 20-35 |
|  | 20d | Present results of all sensitivity analyses conducted to assess the robustness of the synthesized results. | 20-35 |
| Reporting biases | 21 | Present assessments of risk of bias due to missing results (arising from reporting biases) for each synthesis assessed. | 35 |
| Certainty of evidence | 22 | Present assessments of certainty (or confidence) in the body of evidence for each outcome assessed. | 31 |
| **DISCUSSION** | | |  |
| Discussion | 23a | Provide a general interpretation of the results in the context of other evidence. | 35-41 |
|  | 23b | Discuss any limitations of the evidence included in the review. | 41 |
|  | 23c | Discuss any limitations of the review processes used. | 41 |
|  | 23d | Discuss implications of the results for practice, policy, and future research. | 45 |
| **OTHER INFORMATION** | | |  |
| Registration and protocol | 24a | Provide registration information for the review, including register name and registration number, or state that the review was not registered. | 5 |
|  | 24b | Indicate where the review protocol can be accessed, or state that a protocol was not prepared. | 5 |
|  | 24c | Describe and explain any amendments to information provided at registration or in the protocol. | NA |
| Support | 25 | Describe sources of financial or non-financial support for the review, and the role of the funders or sponsors in the review. | 46,47 |
| Competing interests | 26 | Declare any competing interests of review authors. | 46,47 |
| Availability of data, code and other materials | 27 | Report which of the following are publicly available and where they can be found: template data collection forms; data extracted from included studies; data used for all analyses; analytic code; any other materials used in the review. | 46,47 |

*From:* Page MJ, McKenzie JE, Bossuyt PM, Boutron I, Hoffmann TC, Mulrow CD, et al. The PRISMA 2020 statement: an updated guideline for reporting systematic reviews. BMJ 2021;372:n71. doi: 10.1136/bmj.n71

**Supplemental S2-** Research Question, PICOTT and Search Strategy

**Research Question:** Among adult patients undergoing cardiovascular surgery, does perioperative red blood cell transfusion, compared with no transfusion, increase postoperative complications and long-term all-cause mortality, with long-term mortality evaluated through reconstructed time-to-event analysis?

**PICOTT**

**Population (P)**

Adult patients (≥18 years) undergoing **cardiovascular surgery**, including:

- Coronary artery bypass grafting (CABG)
- Valve surgery (aortic, mitral, tricuspid; surgical or rapid-deployment)
- Combined CABG + valve procedures
- Aortic surgery (ascending, arch)
- Other open cardiac surgical procedures requiring cardiopulmonary bypass

**Intervention / Exposure (I)**

**Blood transfusion**, defined as:

- Red blood cell (RBC) transfusion (intraoperative and/or postoperative)

Optional stratification (if data permit):

- Number of RBC units transfused (e.g., 1–2 vs ≥3 units)
- Timing of transfusion (intraoperative vs postoperative)

**Comparator (C)**

**No transfusion**, defined as:

- Patients undergoing cardiovascular surgery **without receipt of RBC transfusion**
- Managed with restrictive or blood-sparing strategies

**Outcomes (O)**

**Primary Outcomes**

- All-cause mortality (in-hospital, 30-day, or long-term)

**Secondary Outcomes**

- Major adverse cardiovascular events (MACE)
- Myocardial infarction
- Stroke / ischemic cerebrovascular events
- Acute kidney injury (AKI)
- Major bleeding or re-operation for bleeding
- Infection / sepsis
- Length of hospital stay
- Intensive care unit (ICU) length of stay

**Timing (T)**

- In-hospital outcomes
- Short-term outcomes (≤30 days)
- Mid-term outcomes (3–12 months)
- Long-term outcomes (≥1 year)

**Type of Studies (T)**

- Randomized controlled trials
- Observational cohort studies
- Registry-based studies
- Propensity score–matched or adjusted analyses

**Subgroup Analyses**

- Type of surgery (CABG vs valve vs combined)
- Transfusion volume (low vs high)
- Timing of transfusion (intraoperative vs postoperative)
- Study design (RCT vs observational vs PSM)

**Search Strategy**

**PubMed search strategy: 721**

("red cell transfusion" OR "packed red blood cell transfusion" OR "Blood cell transfusion" OR "PRBC transfusion") AND ("cardiac surgery" OR "cardiovascular surgery" OR CABG OR "coronary artery bypass graft" OR "valve surgery")

**Embase search strategy: 1654**

("red cell transfusion" OR "packed red blood cell transfusion" OR "Blood cell transfusion" OR "PRBC transfusion") AND ("cardiac surgery" OR "cardiovascular surgery" OR CABG OR "coronary artery bypass graft" OR "valve surgery")

**Scopus search strategy: 962**

("red cell transfusion" OR "packed red blood cell transfusion" OR "Blood cell transfusion" OR "PRBC transfusion") AND ("cardiac surgery" OR "cardiovascular surgery" OR CABG OR "coronary artery bypass graft" OR "valve surgery")

**Cochrane Library: 249**

("red cell transfusion" OR "packed red blood cell transfusion" OR "Blood cell transfusion" OR "PRBC transfusion") AND ("cardiac surgery" OR "cardiovascular surgery" OR CABG OR "coronary artery bypass graft" OR "valve surgery")

**Supplementary S3-** Pooled Kaplan-Meier analysis for overall survival (i.e all-cause mortality). (a) median survival and pooled analysis table (b) Grambsch-Therneau plot (c) hazard ratios over time (d) restricted mean survival time plot (e) funnel plot (f) leave one out analysis

(a) median survival and pooled analysis table

**Median follow-up duration:**

| **Category** | **Median Follow-Up in years (Median [IQR])** |
| --- | --- |
| Overall Survival | 3.13 [1.49, 5.05] |
| No Transfusion | 2.84 [1.43, 4.82] |
| Transfusion | 3.28 [1.49, 5.23] |

**Pooled Analysis:**

| **Strata** | **Time (years)** | **Number at risk** | **Events** | **Survival Probability** | **Standard Error** | **Lower CI** | **Upper CI** |
| --- | --- | --- | --- | --- | --- | --- | --- |
| No Transfusion | 1 | 129830 | 3093 | 0.978331 | 0.000386 | 0.977575 | 0.979087 |
| No Transfusion | 2 | 101240 | 2198 | 0.959584 | 0.000549 | 0.958509 | 0.96066 |
| No Transfusion | 3 | 76983 | 2477 | 0.933153 | 0.000749 | 0.931686 | 0.934622 |
| No Transfusion | 4 | 54883 | 2188 | 0.902498 | 0.000972 | 0.900595 | 0.904406 |
| No Transfusion | 5 | 35899 | 1832 | 0.866523 | 0.00125 | 0.864077 | 0.868976 |
| No Transfusion | 6 | 19318 | 1088 | 0.83262 | 0.001582 | 0.829526 | 0.835727 |
| No Transfusion | 7 | 5572 | 655 | 0.788578 | 0.002351 | 0.783983 | 0.7932 |
| Transfusion | 1 | 83796 | 4560 | 0.950511 | 0.000715 | 0.949111 | 0.951913 |
| Transfusion | 2 | 67342 | 3054 | 0.912175 | 0.000967 | 0.910282 | 0.914071 |
| Transfusion | 3 | 53459 | 2446 | 0.8758 | 0.001176 | 0.873498 | 0.878109 |
| Transfusion | 4 | 40160 | 2573 | 0.828892 | 0.001434 | 0.826086 | 0.831707 |
| Transfusion | 5 | 27965 | 1943 | 0.782271 | 0.001704 | 0.778939 | 0.785617 |
| Transfusion | 6 | 15656 | 1423 | 0.730929 | 0.00208 | 0.726864 | 0.735018 |
| Transfusion | 7 | 4766 | 801 | 0.673148 | 0.002849 | 0.667588 | 0.678755 |

(b) Grambsch-Therneau plot

**Proportionality Hazards ratio:**

**Chi-square: 174, p = 0.0003**

Statistical Violation of PH proportionality assumption.

(c) Hazard ratios over time

(d) restricted mean survival time difference (RMST)

**ΔRMST:**

**Δ at 7 years = -5.37 months; 95% CI (-5.59 to -5.16 months); P < 0.001**

(e) funnel plot

(f) leave one out analysis

| **Omitted Study** | **HR** | **CI_Low** | **CI_High** | **P_Value** | **I^2^** |
| --- | --- | --- | --- | --- | --- |
| Bhasker 2012 | 1.867312 | 1.535275 | 2.27116 | 4.06E-10 | 91.82788 |
| Santos 2013 | 1.880217 | 1.541457 | 2.293424 | 4.69E-10 | 92.33101 |
| Engoren 2009 | 1.898882 | 1.543582 | 2.335965 | 1.3E-09 | 92.0608 |
| Koster 2018 | 2.040009 | 1.835681 | 2.26708 | 5.13E-40 | 64.56377 |
| Kuduvalli 2005 | 1.942464 | 1.588735 | 2.37495 | 9.57E-11 | 92.89894 |
| Schwann 2023 | 1.950727 | 1.579592 | 2.409062 | 5.44E-10 | 84.58285 |
| Sultan 2021 | 1.912299 | 1.548943 | 2.360892 | 1.64E-09 | 88.51819 |
| Sultan 2023 | 1.868434 | 1.549607 | 2.252859 | 5.82E-11 | 91.88788 |
| Tantawy 2018 | 1.965021 | 1.613726 | 2.39279 | 1.79E-11 | 92.57395 |
| Tran 2018 | 1.894277 | 1.550151 | 2.314797 | 4.23E-10 | 92.67433 |

**Supplementary S4-** Forest plots for secondary outcomes. (a) 30-day mortality (pooled odds ratios) (b) long-term mortality (c) atrial fibrillation (d) acute kidney injury (e) myocardial infarction (f) pneumonia (g) reoperation for bleeding (h) sepsis (i) sternal infection (j) stroke

(a) 30-day mortality (pooled odds ratios)

(b) long-term mortality

(c) atrial fibrillation

(d) acute kidney injury

(e) myocardial infarction

(f) pneumonia

(g) reoperation for bleeding

(h) sepsis

(i) sternal infection

(j) stroke

**Supplementary S5**- Forest plots for subgroup analysis based on cardiac surgery type. (a) 30 day mortality (b) 30 day mortality (pooled odds ratio) (c) long term mortality (d) long term mortality (pooled hazard ratios) (e) atrial fibrillation (f) acute kidney injury (g) myocardial infarction (h) pneumonia (i) reoperation for bleeding (j) sepsis (k) sternal infection (l) stroke

(a) 30 day mortality

(b) 30 day mortality (pooled odds ratio)

(c) long term mortality

(d) long term mortality (pooled hazard ratios)

(e) atrial fibrillation

(f) acute kidney injury

(g) myocardial infarction

(h) pneumonia

(i) reoperation for bleeding

(j) sepsis

(k) sternal infection

(l) stroke

**Supplementary S6**- Forest plots for subgroup analysis based on study design. (a) 30 day mortality (b) 30 day mortality (pooled odds ratio) (c) long term mortality (d) long term mortality (pooled hazard ratios) (e) atrial fibrillation (f) acute kidney injury (g) myocardial infarction (h) pneumonia (i) reoperation for bleeding (j) sepsis (k) sternal infection (l) stroke

(a) 30 day mortality

(b) 30 day mortality (pooled odds ratio)

(c) long term mortality

(d) long term mortality (pooled hazard ratios)

(e) atrial fibrillation

(f) acute kidney injury

(g) myocardial infarction

(h) pneumonia

(i) reoperation for bleeding

(j) sepsis

(k) sternal infection

(l) stroke

**Supplementary S7**- Forest plots for subgroup analysis of long term mortality based on transfusion timing and dose. (a) long-term mortality (pooled hazard ratios; dose subgroups) (b) intraoperative transfusion (c) postoperative transfusion (d) intraoperative and postoperative transfusions

(a) long term mortality (pooled hazard ratios; dose subgroups)

(b) intraoperative transfusion

(c) postoperative transfusion

(d) intraoperative and postoperative transfusions

**Supplementary S8-** Funnel plots and egger’s regression test for outcomes**.** (a) 30 day mortality (b) 30 day mortality (pooled odds ratio) (c) long term mortality (d) long term mortality (pooled hazard ratios) (e) atrial fibrillation (f) acute kidney injury (g) myocardial infarction (h) pneumonia (i) reoperation for bleeding (j) sepsis (k) sternal infection (l) stroke

(a) 30 day mortality

β₀ (intercept)	95% CI	t	p

-4.485	-10.37 - 1.4	-1.495	0.273619

Eggers' test does not indicate the presence of funnel plot asymmetry.

(b) 30 day mortality (pooled odds ratio)

β₀ (intercept)	95% CI	t	p

2.009	-1.65 - 5.67	1.075	0.3610823

Eggers' test does not indicate the presence of funnel plot asymmetry.

(c) long-term mortality

β₀ (intercept)	95% CI	t	p

-4.212	-30.73 - 22.3	-0.311	0.8078428

Eggers' test does not indicate the presence of funnel plot asymmetry.

(d) long term mortality (pooled hazard ratios)

β₀ (intercept)	95% CI	t	p

1.898	-0.85 - 4.64	1.356	0.2081782

Eggers' test does not indicate the presence of funnel plot asymmetry.

(e) atrial fibrillation

β₀ (intercept)	95% CI	t	p

0.663	-1.12 - 2.45	0.729	0.5189845

Eggers' test does not indicate the presence of funnel plot asymmetry

(f) acute kidney injury

β₀ (intercept)	95% CI	t	p

3.061	1.8 - 4.32	4.75	0.1321006

Eggers' test does not indicate the presence of funnel plot asymmetry.

(g) myocardial infarction

β₀ (intercept)	95% CI	t	p

1.462	-3.77 - 6.69	0.548	0.6217313

Eggers' test does not indicate the presence of funnel plot asymmetry.

(h) pneumonia

β₀ (intercept)	95% CI	t	p

1.979	-2.1 - 6.05	0.952	0.411385

Eggers' test does not indicate the presence of funnel plot asymmetry.

(i) reoperation for bleeding

β₀ (intercept)	95% CI	t	p

0.663	-1.12 - 2.45	0.729	0.5189845

Eggers' test does not indicate the presence of funnel plot asymmetry.

(j) sepsis

β₀ (intercept)	95% CI	t	p

2.087	-1.68 - 5.85	1.086	0.3909047

Eggers' test does not indicate the presence of funnel plot asymmetry.

(k) sternal infection

β₀ (intercept)	95% CI	t	p

0.117	-1.68 - 1.91	0.127	0.9104335

Eggers' test does not indicate the presence of funnel plot asymmetry.

(l) stroke

β₀ (intercept)	95% CI	t	p

1.074	-0.84 - 2.99	1.1	0.3330313

Eggers' test does not indicate the presence of funnel plot asymmetry.

**Supplementary S9-** Forest plots for leave one out sensitivity analysis. (a) 30 day mortality (b) 30 day mortality (pooled odds ratio) (c) long term mortality (d) long term mortality (pooled hazard ratios) (e) atrial fibrillation (f) acute kidney injury (g) myocardial infarction (h) pneumonia (i) reoperation for bleeding (j) sepsis (k) sternal infection (l) stroke

(a) 30 day mortality

(b) 30 day mortality (pooled odds ratio)

(c) long term mortality

(d) long term mortality (pooled hazard ratios)

(e) atrial fibrillation

(f) acute kidney injury

(g) myocardial infarction

(h) pneumonia

(i) reoperation for bleeding

(j) sepsis

(k) sternal infection

(l) stroke

**Supplementary S10-** Bubble plots for long term mortality (pooled hazard ratios). (a) diabetes mellitus (b) prior myocardial infarction (c) peripheral arterial disease (d) age (e) urgent / emergent surgery (f) female sex

(a) diabetes mellitus

(b) prior myocardial infarction

(c) peripheral arterial disease

(d) age

(e) urgent / emergent surgery

(f) female sex

**Supplementary S11-** Quality assessment using ROBINS - I

**Supplemental S12-** Propensity Score Matching (PSM) Protocol Summary Table

| **Study ID** | **PSM** | **Variables** | **Ratio** |
| --- | --- | --- | --- |
| Ming et al., 2019 | Propensity score matching was performed using a multivariable logistic regression model including perioperative risk factors to reduce confounding when evaluating blood product transfusion outcomes. | **Demographics**   - Gender, ethnicity, age, BMI - Smoking history, alcohol consumption   **Medical history**   - NYHA class - Hypertension - Coronary heart disease - Active endocarditis - Heart failure history - Ejection fraction - Pulmonary hypertension - Stroke - Diabetes mellitus - Renal dysfunction   **Medications**   - Antiplatelets - Anticoagulants - Diuretics   **Preoperative labs**   - Hemoglobin - Platelet count - INR abnormality - Fibrinogen - C-reactive protein   **Surgical characteristics**   - Cardiopulmonary bypass duration - Repeat surgery - Operative procedure | 1:1 |
| Tantawy et al., 2018 | Patients receiving transfusion were matched to non-transfused patients using propensity score matching to correct baseline characteristic bias | **Demographics**   - Age, sex, BMI   **Comorbidities**   - Diabetes - Dialysis - Hypertension - Peripheral arterial disease - Cerebrovascular disease - Prior MI - Recent heart failure - Anticoagulant use - Left main disease - Cardiogenic shock - Number of diseased vessels   **Laboratory**   - Creatinine - Hemoglobin before surgery - Ejection fraction   **Surgical characteristics**   - Surgery urgency (elective/urgent/emergent/salvage) - Year of surgery | 1:1 |
| Sultan et al., 2023 | Propensity matching used to generate equivalent cohorts due to baseline differences. | Sex, Age, Ejection fraction, Hematocrit, Total albumin, Diabetes, Dialysis, COPD, Dyslipidemia, Previous cardiac surgery, Preoperative anticoagulation, Preoperative antiplatelet use, Surgical status, Dissection presence, Hemiarch vs total arch, Surgery type | 1:1 |
| Koster et al., 2018 | Propensity score matching used due to non-randomized group assignment to evaluate transfusion effect. | Age, Sex, BMI, Type of cardiac surgery, Cardiopulmonary bypass use, Estimated GFR, EuroSCORE, Diabetes mellitus, Smoking, Cardiac reoperation, Preoperative anemia, Aspirin + clopidogrel use | 1:1 |
| Bhaskar et al., 2012 | Propensity score constructed using logistic regression from core patient characteristics (46 variables) to control treatment selection bias. | 46 perioperative patient characteristics (not individually listed in detail). | Each transfused patient matched with a non-transfused patient using identical propensity scores within 1%.  (Effectively ~1:1 matching.) |
| Sultan et al., 2021 | Propensity-matched cardiac surgery patients | Age, Sex (female), Race, BMI, Hypertension, Dyslipidemia, Diabetes mellitus, Dialysis, Peripheral vascular disease, Cerebrovascular disease, Previous MI, Immunosuppression, Previous heart failure, COPD, Family history of CAD, Previous CABG, Previous valve surgery, NYHA class, No angina, Non-ischemic symptoms, Stable angina, Unstable angina, NSTEMI, STEMI, Angina equivalent, Other, Operative status: Elective, Urgent, Emergent, Operation category: Simple, Double valve, aortic root, Triple valve, CABG procedure presence, Preoperative IABP, Antiplatelet within 5 days, Anticoagulant within 5 days, LVEF (%), Total albumin, Hemoglobin, Hematocrit, Serum creatinine | 1:1 |
| Vlot et al., 2019 | Used propensity-score–based logistic regression adjustment, not matching. | Age, sex, BMI, Preop hemoglobin, Creatinine, Smoking, COPD, Hypertension, Diabetes, Peripheral artery disease, Previous stroke, LVEF, Previous cardiac surgery, Unstable angina, Emergency surgery, Duration of operation, Autologous RBC transfusion, Internal mammary artery use, Type of surgery | NA |
| Kuduvalli et al., 2005 | Propensity score = probability of receiving transfusion based on clinical characteristics. | Lowest hemoglobin, BMI, Cardiopulmonary bypass use, Female sex, Number of grafts, Renal dysfunction  Age, Disease extent, Prior CABG | NA |
| Engoren et al., 2009 | Used propensity analysis to control for sicker patients receiving transfusion | NA | NA |

LVEF: Left ventricular ejection fraction, BMI: Basal metabolic index, CABG: Coronary artery bypass graft, NA: Not applicable, COPD: Chronic obstructive pulmonary disease, NYHA: New York Heart Association, RBC: Red blood cell, GFR: Glomerular filtration rate, INR: International normalized ratio, IABP: Intraaortic balloon pump, CAD: Coronary artery disease, MI: Myocardial infarction, EuroSCORE: European system for cardiac operative risk evaluation, STEMI: ST segment elevation myocardial infarction, NSTEMI: Non ST segment elevation myocardial infarction.
